# Innate immune circuits in acute lung injury: macrophage plasticity, ILC crosstalk, and tissue repair failure

**DOI:** 10.3389/fimmu.2026.1878164

**Published:** 2026-06-23

**Authors:** Xiaoya Wang, Xiaolin Wang, Li Li, Li Wang, Kun Fang

**Affiliations:** 1 Department of Pathology, Affiliated Hospital of North Sichuan Medical College, Nanchong, China; 2 Institute of Basic Medicine, North Sichuan Medical College, Nanchong, China; 3 Department of Surgery, Yinchuan Maternal and Child Health Hospital, Yinchuan, China

**Keywords:** acute lung injury, acute respiratory distress syndrome, innate lymphoid cells, macrophage plasticity, tissue repair failure

## Abstract

Acute lung injury (ALI) and acute respiratory distress syndrome (ARDS) are traditionally understood as hyperinflammatory syndromes characterized by cytokine excess, neutrophil infiltration, and disruption of the alveolar–capillary barrier. However, this framework does not fully explain why some injured lungs undergo effective resolution whereas others progress toward persistent inflammation, defective epithelial regeneration, and long-term pulmonary dysfunction. Increasing evidence suggests that ALI is better conceptualized as a disorder of dysregulated tissue-centered innate immune circuits rather than a simple consequence of uncontrolled inflammation. Among the key cellular regulators of these circuits, macrophages and innate lymphoid cells (ILCs), especially ILC2s, play central and complementary roles. Macrophages function as early sentinels, inflammatory amplifiers, efferocytic cleaners, and reparative coordinators, with their impact determined by lineage origin, temporal state transitions, and niche-dependent plasticity. ILCs, in parallel, translate epithelial alarm signals into tissue-adaptive responses and contribute to barrier protection, homeostatic restoration, and modulation of macrophage function. Importantly, macrophages, ILCs, and epithelial cells form an interdependent communication network that governs the balance between inflammatory escalation and successful repair. When this network becomes disrupted, the injured lung shifts from coordinated recovery to failed repair. In this review, we discuss macrophage heterogeneity and plasticity in ALI, epithelial–macrophage and macrophage–ILC crosstalk, mechanisms of inflammatory resolution and repair failure, and emerging therapeutic opportunities aimed at restoring innate immune circuit competence in the injured lung.

## Highlights

- ALI should be understood as a disorder of dysregulated lung innate immune circuits rather than uncontrolled inflammation alone.
- Macrophage plasticity and ILC-mediated tissue responses jointly determine the balance between inflammatory amplification and repair.
- Restoring macrophage–ILC–epithelial circuit competence may provide new therapeutic opportunities for ALI.

## 1 Introduction

Acute lung injury (ALI) and its more severe clinical manifestation, acute respiratory distress syndrome (ARDS), represent major causes of respiratory failure in critically ill patients (1–4). They can be triggered by diverse insults, including severe infection, sepsis, pneumonia, aspiration, mechanical ventilation, and sterile tissue injury. Despite this etiologic heterogeneity, these conditions converge on a shared pathological phenotype characterized by diffuse inflammatory damage within the lung, disruption of alveolar–capillary barrier integrity, pulmonary edema, impaired gas exchange, and, in severe cases, life-threatening hypoxemia (5–7). Traditional models of ALI have largely framed the disease as an acute hyperinflammatory syndrome driven by excessive cytokine production, endothelial and epithelial barrier breakdown, and massive recruitment of neutrophils into the alveolar space (8, 9). This classical view has undoubtedly provided an important foundation for understanding ALI pathogenesis. Elevated inflammatory mediators, alveolar flooding, oxidative stress, protease release, and neutrophil-dependent tissue injury remain central components of disease progression. However, this framework is no longer sufficient to explain the full biological complexity of ALI. In particular, it does not adequately account for the marked divergence in clinical and pathological outcomes among patients exposed to seemingly similar insults. Some individuals undergo effective inflammatory resolution, restore epithelial integrity, and recover pulmonary function, whereas others develop persistent inflammation, defective alveolar repair, prolonged ventilator dependence, fibroproliferative remodeling, or long-term respiratory sequelae (10–13). These differences suggest that ALI cannot be understood solely as a matter of inflammatory intensity. A more informative perspective is to view ALI as a dynamic failure of tissue-level immune regulation. The injured lung is not merely a passive target of circulating inflammatory mediators; rather, it is an active immunological niche in which epithelial cells, resident and recruited innate immune cells, stromal components, and local metabolic cues continuously interact. Within this context, disease progression depends not only on the initiation of inflammation, but also on how effectively the lung coordinates damage sensing, inflammatory amplification, cellular clearance, barrier restoration, and return to homeostasis (14, 15). When these processes become uncoupled in time or distorted in magnitude, acute inflammation can transition into persistent tissue dysfunction and failed repair. Accordingly, ALI should be conceptualized less as a simple cytokine-driven storm and more as a disorder of dysregulated local immune circuits operating within the pulmonary microenvironment. This shift in perspective is particularly important in the current era, as emerging studies increasingly highlight the role of cellular heterogeneity, tissue-resident immune programs, intercellular communication, and context-dependent repair responses in shaping lung injury outcomes (16–18). Understanding why inflammation resolves in some settings but becomes self-sustaining in others requires a framework that integrates immune effector function with tissue ecology. Such a framework is especially relevant for identifying the mechanisms that link acute injury to impaired regeneration and for developing therapies aimed not only at suppressing inflammation, but also at restoring coordinated tissue recovery.

Among the many cellular players involved in ALI, macrophages and innate lymphoid cells (ILCs) occupy especially important positions because they sit at the interface between early injury sensing, inflammatory orchestration, and tissue repair (19, 20). Macrophages are among the most functionally versatile innate immune cells in the lung (21, 22). Under homeostatic conditions, resident alveolar macrophages help maintain immune quiescence, remove inhaled particles, clear apoptotic cells, and preserve alveolar integrity. During acute injury, however, macrophage populations rapidly undergo phenotypic and functional remodeling. They can detect pathogen-associated or damage-associated signals, produce inflammatory cytokines and chemokines, recruit additional leukocytes, and amplify tissue injury (16, 23, 24). At the same time, macrophages are also indispensable for the resolution phase, where they mediate efferocytosis, limit excessive inflammation, support epithelial regeneration, and help re-establish local homeostasis (21, 25–27). Thus, macrophages are not simply inflammatory effectors; they are central regulators of the transition from damage to repair. Importantly, macrophage function in ALI cannot be captured by static or overly simplified classifications. The lung contains both tissue-resident alveolar macrophages and recruited monocyte-derived macrophages, and these populations may display distinct, overlapping, or shifting roles depending on the phase of injury, anatomical niche, and local signaling milieu. Their behavior is shaped by epithelial-derived factors, metabolic stress, extracellular vesicles, inflammatory mediators, and interactions with other immune cells. For this reason, macrophage heterogeneity and plasticity are not peripheral details, but core determinants of whether the injured lung undergoes inflammatory escalation or coordinated recovery. ILCs, particularly group 2 innate lymphoid cells (ILC2s), provide a complementary and equally important dimension to this picture (28, 29). As tissue-resident lymphoid cells lacking antigen-specific receptors, ILCs are strategically positioned to respond rapidly to epithelial alarm signals and environmental perturbations. In the lung, ILC2s contribute to mucosal homeostasis, barrier support, and wound-healing responses through the production of type 2 cytokines, amphiregulin, and other tissue-reparative mediators (30). Although they are often discussed primarily in the context of allergic inflammation, accumulating evidence indicates that their biological roles extend well beyond classic type 2 immunity. In settings of acute pulmonary injury, ILC2s can participate in epithelial protection, tissue restoration, and the re-establishment of local equilibrium. At the same time, under certain inflammatory conditions, their activation may also contribute to maladaptive remodeling or pathological immune bias. Focusing on macrophages and ILCs together is especially valuable because these cells do not operate in isolation. Instead, they are embedded in interconnected communication networks shaped by cytokines, growth factors, lipid mediators, metabolic signals, and structural cell-derived cues. Epithelial cells can simultaneously condition macrophage activation and ILC responsiveness; macrophages can influence ILC recruitment, polarization, and function; and ILC-derived mediators can, in turn, modify macrophage behavior and reparative capacity (31). These multidirectional interactions suggest that the key unit of pathogenesis is not an individual cell type, but a coordinated innate immune circuit. Examining macrophages and ILCs within the same conceptual framework therefore offers a more integrated understanding of how the lung balances inflammatory defense with tissue preservation, and why this balance fails in severe ALI.

In this review, we examine ALI as a disorder of tissue-centered innate immune regulation, focusing on macrophage plasticity, ILC-mediated responses, and immune–structural cell communication. We first discuss the heterogeneity of resident alveolar macrophages and recruited monocyte-derived macrophages, emphasizing their stage-dependent roles in inflammation, efferocytosis, and repair. We then examine epithelial–macrophage interactions and how epithelial alarm signals, extracellular vesicles, growth factors, and niche-derived cues shape innate immune responses. Next, we discuss ILCs, particularly ILC2s, as context-dependent regulators of epithelial protection, tissue adaptation, and repair support. Building on these concepts, we consider macrophage–ILC crosstalk as an emerging framework for understanding how innate immune networks may influence inflammatory amplification, resolution, and repair failure. Finally, we summarize mechanisms of failed repair and discuss therapeutic opportunities and translational limitations of targeting innate immune circuits in ALI.

## 2 The lung as a tissue-specialized innate immune niche in ALI

### 2.1 The alveolar niche shapes innate immune responses

The lung is not simply a target organ in acute lung injury, but a highly specialized immunological environment in which structural and immune cells are tightly integrated. Nowhere is this more evident than in the alveolar compartment, where the requirements for rapid host defense must be balanced against the need to preserve an exceptionally delicate gas-exchange surface. Under physiological conditions, this balance is maintained through continuous communication between alveolar epithelial cells, resident immune cells, endothelial cells, and local stromal elements (32–36). These interactions establish a tissue niche that does not merely support immune cell residence, but actively instructs immune cell identity, threshold of activation, and functional output.

Within this niche, alveolar macrophages are particularly important. Unlike inflammatory myeloid cells recruited from the circulation during injury, resident alveolar macrophages are long adapted to the pulmonary environment and are conditioned by local signals to perform homeostatic functions with minimal collateral damage (37). They clear inhaled particles, remove dying cells, restrain unnecessary inflammation, and contribute to surfactant-associated equilibrium. Their phenotype and survival, however, are not cell-autonomous. Instead, they are sustained by niche-derived cues provided largely by the alveolar epithelium. This point is crucial, because it means that macrophage behavior in ALI is inseparable from the condition of the surrounding tissue. Once the epithelial niche is perturbed, macrophage programs are also redefined.

The alveolar epithelium, especially type II alveolar epithelial cells, plays a central role in this niche organization (38–41). These cells do far more than provide structural support or participate in surfactant metabolism. They function as local immune regulators by delivering trophic and instructional signals that preserve tissue-resident macrophage identity. In this way, structural cells act as gatekeepers of innate immunity. The implication for ALI is significant: injury to the epithelium does not only compromise barrier integrity, but also destabilizes the very signaling networks that maintain macrophage homeostasis. As a result, the inflammatory behavior of macrophages in acute injury cannot be explained solely by exogenous stimuli such as pathogens or circulating cytokines; it also reflects disruption of the local ecological context that normally constrains and guides innate immune function. This niche-centered view also helps explain why pulmonary innate immunity is so context dependent. The same macrophage population may exhibit markedly different behavior depending on the availability of epithelial-derived growth factors, metabolic substrates, extracellular vesicles, and local alarmins. Likewise, the transition from homeostatic surveillance to inflammatory amplification is not merely a switch turned on by injury, but a progressive reprogramming event shaped by the changing tissue environment. This concept is particularly relevant in ALI, where alveolar flooding, epithelial death, oxidative stress, and altered mechanical forces rapidly remodel the pulmonary niche (42, 43). In such settings, the lung becomes a permissive environment for innate immune dysregulation, and tissue damage is amplified not only by recruited inflammatory cells but also by the malfunction of resident immune–structural partnerships. Importantly, the alveolar niche is not static during injury and recovery. In the early phase of ALI, epithelial disruption and endothelial leakage alter the biochemical and cellular composition of the alveolar space. During the resolution phase, restoration of niche signals becomes a prerequisite for effective repair, because reparative macrophage programs depend on a supportive tissue context. Thus, the lung microenvironment should be viewed as an active determinant of disease trajectory, influencing whether innate immune cells remain destructive, convert to pro-resolving states, or fail to complete the transition toward tissue restoration. Framing ALI in this way shifts emphasis from inflammatory burden alone to the integrity of the tissue ecosystem in which inflammation unfolds.

### 2.2 Etiological triggers of ALI and injury-induced epithelial alarm signaling

ALI can be initiated by a wide range of direct and indirect insults that converge on disruption of the alveolar–capillary barrier (44). Direct pulmonary causes include bacterial or viral pneumonia, aspiration of gastric contents, inhalational injury, toxic exposure, pulmonary contusion, and ventilator-induced lung injury (1, 21, 45). Indirect systemic causes include sepsis, severe trauma, shock, pancreatitis, burns, transfusion-related acute lung injury, ischemia–reperfusion injury, and systemic inflammatory states (46, 47). Although these triggers differ in origin, they share several downstream pathogenic features, including epithelial and endothelial injury, increased vascular permeability, alveolar edema, neutrophil recruitment, oxidative stress, coagulation activation, and release of pathogen-associated or damage-associated molecular patterns. Therefore, ALI should not be understood as a single-cause disease, but as a convergent syndrome in which diverse injurious stimuli collapse the structural and immunological integrity of the alveolar niche. The biological consequences of these triggers are strongly shaped by host and microenvironmental factors. Aging, obesity, metabolic dysfunction, pre-existing lung disease, impaired epithelial regeneration, endothelial vulnerability, defective efferocytosis, and persistent neutrophil activation may all influence whether an initial insult resolves or progresses toward sustained inflammation and repair failure (27, 48, 49). Mechanical ventilation, although often life-saving, can further amplify injury through volutrauma, barotrauma, atelectrauma, and mechanotransduction-dependent inflammatory signaling. Thus, the severity and persistence of ALI reflect not only the nature of the initiating insult, but also the susceptibility of the alveolar niche and the capacity of local immune circuits to restore homeostasis.

When acute lung injury begins, epithelial cells are among the first local sensors and transmitters of danger (50–52). Their role is not limited to being damaged targets of infection, toxic exposure, or mechanical stress. Instead, injured epithelial cells actively initiate and shape innate immune responses by releasing a broad range of alarm signals that communicate tissue distress to surrounding immune populations. These signals include cytokines, growth factors, extracellular vesicles, metabolites, and danger-associated molecular patterns, all of which help transform a localized insult into an organized inflammatory and reparative response. In this sense, epithelial cells serve as upstream regulators of innate immune circuit activation in the injured lung. This idea is particularly important for understanding why ALI evolves differently across patients and experimental contexts. Classical descriptions of lung injury often focus on downstream outcomes such as neutrophil accumulation, cytokine release, and edema formation, but the upstream pattern of epithelial signaling may be just as decisive (53–55). Depending on the nature and severity of injury, epithelial-derived cues can promote inflammatory recruitment, activate resident macrophages, bias incoming monocytes toward pathogenic phenotypes, and stimulate ILC responses linked to tissue adaptation or repair. Thus, the injured epithelium is not a passive barrier undergoing destruction, but an active command center that helps determine whether innate immunity becomes coordinated or dysregulated. Among the most relevant consequences of epithelial injury is the altered dialogue with macrophages. Damaged epithelial cells can release extracellular vesicles that carry inflammatory signals capable of activating macrophages and amplifying tissue injury (56). They can also induce macrophages to produce trophic mediators that influence epithelial proliferation and repair. This bidirectional communication illustrates a central principle of ALI pathogenesis: inflammation and repair are not sequentially isolated events, but overlapping processes coordinated by reciprocal signaling between structural and immune cells. When epithelial alarm programs are excessive or prolonged, macrophages may be driven toward persistent inflammatory activity. Conversely, when epithelial–macrophage communication is appropriately rebalanced, it may support the emergence of pro-resolving and reparative macrophage states. Epithelial signals also have major implications for ILC biology. Innate lymphoid cells, particularly ILC2s, are exquisitely responsive to tissue-derived mediators released during epithelial stress. Rather than requiring antigen-specific priming, they respond rapidly to local perturbation and are therefore well positioned to translate epithelial damage into broader tissue-level adaptation. In the setting of acute injury, this may allow ILC2s to contribute to barrier support, epithelial survival, and restoration of homeostasis. However, these same pathways may also become maladaptive when injury is severe, repetitive, or unresolved. As a result, epithelial-ILC signaling should be viewed as a context-dependent axis that may either reinforce tissue protection or contribute to persistent remodeling.

**Figure 1:**
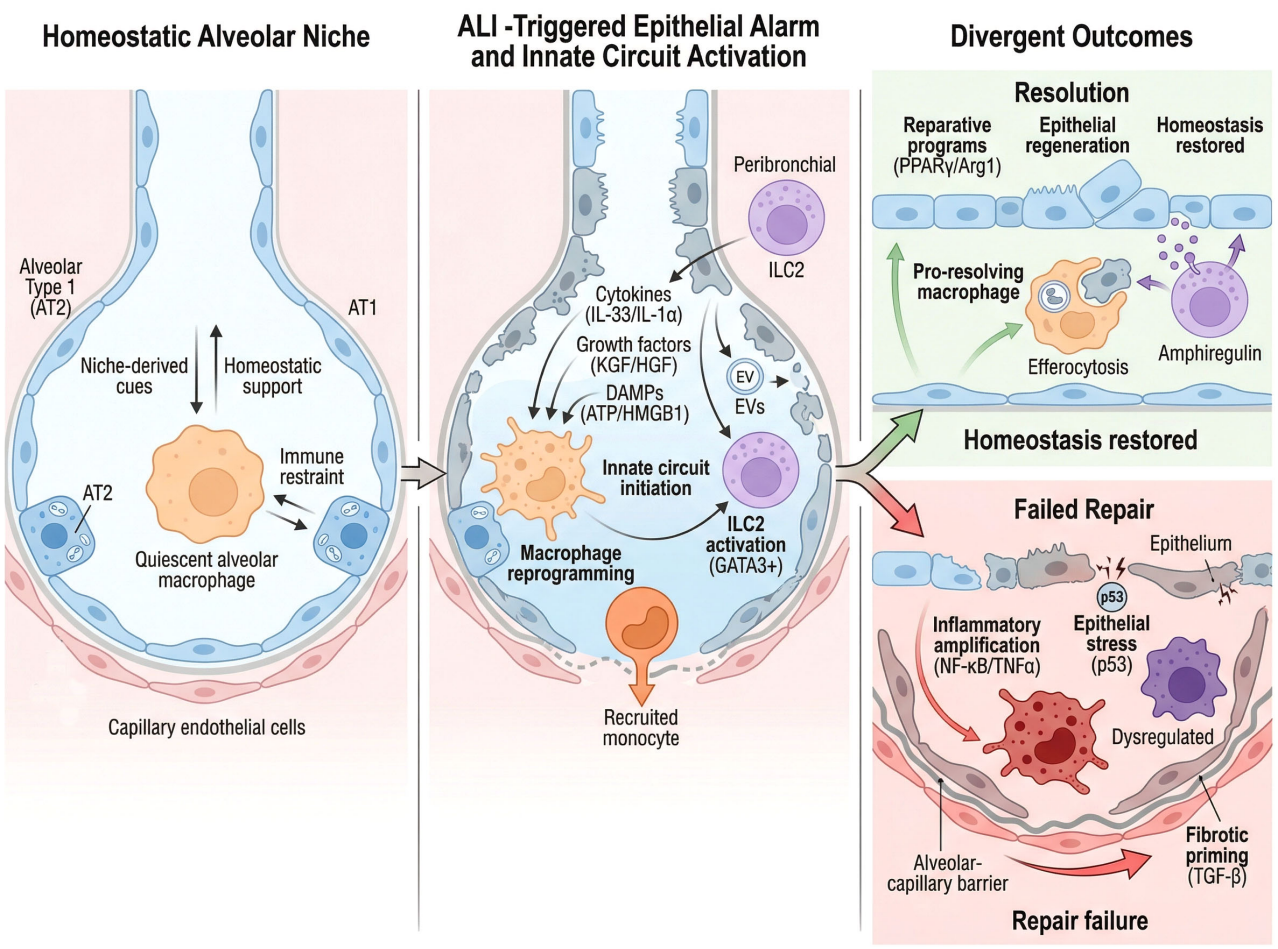
Epithelial-centered innate immune circuits in acute lung injury. In ALI, injured alveolar epithelial cells act as upstream organizers by releasing alarm signals that activate resident macrophages, recruit inflammatory monocyte-derived macrophages, and stimulate ILC2 responses. These epithelial-driven circuits determine whether the lung undergoes coordinated resolution and epithelial repair or progresses toward persistent inflammation, disrupted immune crosstalk, and failed tissue restoration.

Taken together, these observations support a model in which ALI begins not simply with inflammatory cell infiltration, but with the collapse and reprogramming of epithelial-centered communication networks. The subsequent behavior of macrophages, ILCs, and other innate immune cells is shaped by the nature of these early tissue-derived signals. This concept has important mechanistic and therapeutic implications. It suggests that the determinants of disease severity may lie not only in the magnitude of downstream inflammation, but also in how the injured epithelium encodes and transmits danger, repair, and resolution signals to surrounding immune cells. Accordingly, defining epithelial alarm programs and their downstream innate circuits is essential for understanding why some injured lungs successfully return to homeostasis whereas others enter a state of persistent inflammation and failed repair. **Figure 1** illustrates that ALI is not merely a consequence of uncontrolled inflammation, but a disorder of epithelial-centered innate immune circuit dysregulation involving macrophages, ILC2s, and the alveolar niche.

## 3 Macrophage plasticity in acute lung injury

### 3.1 Development, niche maintenance, and homeostatic functions of alveolar macrophages

A deeper understanding of alveolar macrophage biology is essential before their pathogenic roles in ALI can be interpreted (16). Resident alveolar macrophages are not simply generic tissue macrophages located in the alveolar space; they represent a highly specialized macrophage population adapted to the unique requirements of the gas-exchange surface. Under physiological conditions, AMs are established during early lung development and are subsequently maintained largely through local self-renewal rather than continuous replacement by circulating monocytes (57, 58). Their identity and survival depend on alveolar niche-derived signals, particularly epithelial cell-derived GM-CSF/CSF2, together with additional local cues such as TGF-β, surfactant-associated lipids, and the transcriptional program centered on PPARγ. These signals enable AMs to acquire a phenotype suited to the alveolar environment, where immune competence must be balanced with strict restraint of unnecessary inflammation.

In the healthy lung, AMs perform several nonredundant homeostatic functions. They clear excess surfactant and inhaled particles, remove dying cells, provide basal immune surveillance, and maintain a low inflammatory tone compatible with efficient gas exchange (59, 60). This homeostatic restraint is biologically important because even modest inflammatory activation within the alveolar compartment can compromise epithelial and endothelial barrier integrity. Therefore, AMs should be viewed not only as immune sentinels but also as niche-stabilizing cells that preserve pulmonary equilibrium. Disruption of this homeostatic program may represent an early and underappreciated step in ALI pathogenesis (2). Once epithelial injury, surfactant dysfunction, oxidative stress, mechanical stretch, or inflammatory cytokines perturb the alveolar niche, AMs may lose the local instructions that normally sustain their identity and restrained activation state (61, 62). In this sense, ALI involves not only activation of AMs but also destabilization of the niche-dependent programs that normally maintain AM-mediated pulmonary homeostasis.

This perspective also clarifies why AM responses in ALI are highly context dependent. A resident AM exposed to transient epithelial stress may initiate controlled inflammatory signaling while preserving the capacity to return to a pro-resolving state. In contrast, severe or persistent injury may disrupt the epithelial–AM niche, impair surfactant handling, reduce efferocytic competence, and promote sustained cytokine production. Thus, the pathogenic role of AMs in ALI is not simply that they become “pro-inflammatory”; rather, they may lose the capacity to coordinate homeostatic surveillance, inflammatory containment, debris clearance, and repair in a temporally ordered manner. Recognizing this biology provides a more mechanistic basis for understanding how AM dysfunction contributes to the transition from reversible acute inflammation to persistent tissue injury and failed repair.

### 3.2 Resident alveolar macrophages as early sentinels and amplifiers during ALI

Positioned directly within the alveolar space, they are strategically located to sense microbial products, inhaled toxins, mechanical stress, surfactant alterations, and signals released by injured epithelial cells (63, 64). This anatomical positioning makes them indispensable for early host defense, but it also means that they are uniquely capable of initiating inflammatory cascades at the earliest stages of lung injury. In ALI, therefore, alveolar macrophages are not merely downstream responders recruited after tissue damage has begun; they are frontline sentinels that can help set the tone of the entire local immune response.

Resident AMs and recruited monocyte-derived macrophages should be distinguished not only by anatomical location or surface markers, but also by developmental origin, niche dependence, activation threshold, and functional trajectory during injury (65, 66). Resident AMs are long adapted to the alveolar niche and normally support surfactant homeostasis, immune quiescence, and controlled surveillance. By contrast, recruited monocyte-derived macrophages enter the lung in response to chemokine gradients and inflammatory vascular activation, and their differentiation occurs within a tissue environment already enriched in epithelial damage signals, cytokines, hypoxia, and metabolic stress (67, 68). As a result, these cells often display more inflammatory and less niche-adapted phenotypes during early ALI. However, they should not be viewed as uniformly pathogenic, because under specific conditions recruited macrophages may also acquire clearance-associated or reparative functions. The key unresolved question is therefore not whether recruited macrophages are “bad” and resident AMs are “good,” but how the balance, timing, and reprogramming of these two macrophage pools determine whether ALI progresses or resolves.

Under homeostatic conditions, alveolar macrophages typically display a restrained functional profile adapted to the fragility of the gas-exchange interface (22). Excessive activation would be incompatible with normal alveolar function, so these cells are ordinarily tuned toward immune tolerance, debris clearance, and low-level surveillance. Acute injury disrupts this balance. Once activated by damage-associated cues or epithelial distress signals, resident alveolar macrophages can rapidly shift toward a pro-inflammatory state characterized by the production of cytokines, chemokines, proteases, and other mediators that recruit additional leukocytes and intensify local inflammation (22, 60, 65, 69). In this setting, their early response can be beneficial for pathogen containment or danger recognition, but when excessive or poorly resolved, it can become a major driver of alveolar barrier dysfunction.

This duality is central to understanding macrophage plasticity in ALI. The same resident macrophage population that normally sustains tissue equilibrium may become a potent amplifier of injury under conditions of severe stress. Experimental evidence has shown that alveolar macrophages contribute to disruption of the alveolar barrier during ventilator-induced lung injury, supporting the idea that these cells can directly worsen pulmonary permeability rather than simply responding to damage that has already occurred (70). Likewise, macrophage-derived extracellular vesicles and microvesicles can act as paracrine effectors that propagate inflammatory signals within the alveolar microenvironment (71). These findings are important because they broaden the concept of macrophage-mediated injury beyond direct cytokine secretion alone, emphasizing that alveolar macrophages participate in a broader communication network that shapes epithelial and endothelial dysfunction.

Another important feature of resident alveolar macrophages in early ALI is that their activity is tightly conditioned by the surrounding tissue context. They do not become pathogenic in isolation. Instead, their functional shift is driven by epithelial injury, altered surfactant composition, oxidative stress, mechanical strain, and changing metabolic conditions within the alveolar space. This contextual dependence helps explain why similar macrophage populations may behave differently across distinct forms of lung injury, such as infection-driven ALI, sepsis-associated lung injury, or ventilator-induced damage. It also reinforces the idea that macrophage activation should be understood as a tissue-embedded process rather than a cell-intrinsic binary switch. At the same time, resident alveolar macrophages should not be viewed solely as harmful initiators of inflammation. Their early activation may also influence how efficiently the injured lung transitions toward subsequent control and repair. In some contexts, timely macrophage sensing and controlled inflammatory signaling may help orchestrate appropriate leukocyte recruitment and pathogen clearance (72). The problem arises when early macrophage activation is excessive, sustained, or disconnected from later pro-resolving programs. In such circumstances, cells that were initially deployed as local sentinels become contributors to self-perpetuating tissue injury. Thus, the pathogenic significance of resident alveolar macrophages in ALI lies not only in their capacity to trigger inflammation, but also in the possibility that they fail to disengage from inflammatory amplification once the initial insult has occurred.

For this reason, alveolar macrophages should be conceptualized as early gatekeepers of disease trajectory. Their response to injury may influence whether the lung enters a phase of controlled, self-limited inflammation or progresses toward escalating barrier failure and broader innate immune dysregulation. Understanding the factors that govern this initial macrophage transition is therefore essential for interpreting ALI as a dynamic circuit disorder rather than a static inflammatory event.

### 3.3 Recruited monocyte-derived macrophages and inflammatory escalation

Although resident alveolar macrophages dominate the earliest phase of local danger sensing, they are not the only macrophage population that shapes acute lung injury (73, 74). As tissue injury progresses, circulating monocytes are recruited into the lung and differentiate into monocyte-derived macrophages, thereby adding a second and highly consequential myeloid compartment to the injured pulmonary microenvironment (36, 75). These recruited cells do not simply increase the total macrophage burden. Rather, they introduce new inflammatory programs, alter intercellular communication networks, and may fundamentally reshape the trajectory of ALI by reinforcing tissue damage or, under certain conditions, participating in later repair.

The entry of monocyte-derived macrophages into the injured lung reflects the transition from immediate local sensing to a broader inflammatory phase. Once alveolar epithelial cells, resident macrophages, and endothelial cells release chemotactic and inflammatory mediators, the pulmonary niche becomes permissive for monocyte influx (76–79). In many forms of ALI and ARDS, this recruited compartment is associated with heightened inflammatory activity, increased cytokine production, and amplification of tissue injury. This is particularly important because recruited macrophages are often less constrained by the homeostatic conditioning that characterizes resident alveolar macrophages. Having differentiated in the setting of acute inflammation, they may display phenotypes that are more overtly inflammatory, more metabolically stressed, and more tightly coupled to ongoing tissue destruction.

Emerging evidence indicates that these monocyte-derived macrophages are not passive supplements to resident cells, but active participants in immune dysregulation (80). Single-cell profiling in ARDS has identified early monocyte-associated transcriptional programs linked to inflammatory activation, supporting the concept that macrophage pathogenicity in acute lung injury cannot be reduced to the behavior of alveolar macrophages alone (81). Instead, ALI should be understood as involving dynamic interactions between tissue-resident and recruited myeloid compartments, each shaped by distinct developmental origins, signaling histories, and environmental constraints. This distinction is biologically significant because resident and recruited macrophages may occupy different niches, respond differently to epithelial damage, and exert divergent effects on endothelial integrity, leukocyte recruitment, and inflammatory persistence.

The relationship between these two macrophage pools is especially relevant to disease progression. In severe injury, recruited monocyte-derived macrophages may outcompete, displace, or functionally override resident alveolar macrophages, thereby shifting the pulmonary immune environment away from controlled surveillance and toward sustained inflammatory escalation. Recent evidence suggests that inflammatory monocyte-derived macrophages can even promote the death of resident alveolar macrophages through cytokine-dependent mechanisms, highlighting that macrophage populations in ALI may not merely coexist but actively reshape one another (74, 82, 83). Such findings add another layer of complexity to the concept of macrophage plasticity: pathogenesis is influenced not only by state transitions within a given macrophage subset, but also by alterations in the balance between distinct macrophage lineages. This recruited compartment is also highly relevant to the concept of tissue-driven inflammation. Once monocytes enter the injured lung, their differentiation is strongly conditioned by the local niche. Signals derived from injured epithelium, activated endothelium, extracellular vesicles, metabolic stress, and resident immune cells all contribute to defining whether monocyte-derived macrophages remain predominantly inflammatory or begin to acquire functions related to clearance and repair. Thus, monocyte recruitment should not be viewed as a fixed pathogenic event, but as the beginning of a context-dependent reprogramming process. Nevertheless, when the inflammatory milieu remains dominant, this compartment can become a major source of persistent cytokine production, endothelial dysfunction, and failure of inflammatory termination. For these reasons, recruited monocyte-derived macrophages represent a pivotal bridge between acute injury initiation and sustained immunopathology. Their accumulation marks a shift from localized sensing to system-level inflammatory amplification within the lung. More importantly, their persistence may help explain why some cases of ALI do not resolve efficiently even after the initial insult has been controlled. Understanding how these cells are recruited, instructed, and potentially redirected toward reparative states is therefore central to any tissue-based model of ALI progression. Rather than treating macrophages as a single pool with variable activation status, a more accurate framework recognizes that the interplay between resident alveolar macrophages and recruited monocyte-derived macrophages is itself a core determinant of whether lung injury escalates or begins to resolve.

### 3.4 Beyond M1/M2: dynamic states, timing, and functional rewiring

The M1/M2 paradigm has long been used as a simplified framework to describe macrophage polarization (84, 85). Classically activated M1-like macrophages are generally induced by microbial products and type 1 inflammatory signals, such as lipopolysaccharide and interferon-γ, and are associated with the production of pro-inflammatory mediators, including TNF-α, IL-1β, IL-6, inducible nitric oxide synthase, and chemokines that promote leukocyte recruitment (86, 87). In contrast, alternatively activated M2-like macrophages are typically induced by type 2 cytokines such as IL-4 and IL-13 and are linked to anti-inflammatory regulation, efferocytosis, extracellular matrix remodeling, angiogenesis, and tissue repair. This classification has been useful for introducing broad functional differences between inflammatory and reparative macrophage programs (21, 88). The complexity of macrophage behavior in acute lung injury cannot be adequately captured by the classical M1/M2 paradigm. Although this binary framework has historically provided a convenient language for describing pro-inflammatory versus anti-inflammatory macrophage polarization, it is increasingly clear that such a model is overly reductionist in the context of tissue injury (89–91). In ALI, macrophages do not simply toggle between two discrete and stable states. Instead, they occupy a continuum of temporally and spatially regulated phenotypes shaped by developmental origin, tissue niche, inflammatory intensity, metabolic stress, and interactions with epithelial, endothelial, and lymphoid cells. Appreciating this complexity is essential for understanding why macrophages can contribute to both injury propagation and recovery within the same disease process.

One limitation of the M1/M2 framework is that it tends to abstract macrophages away from the tissue environment in which they operate. In the injured lung, however, macrophage function is inseparable from local context. Resident alveolar macrophages, newly recruited monocyte-derived macrophages, and transitional intermediates may all coexist during ALI, yet they do not respond identically to the same signals. Their phenotypes are shaped not only by canonical inflammatory cytokines, but also by extracellular vesicles, hypoxic stress, oxidative injury, dying cell burden, epithelial-derived growth factors, surfactant alterations, and the evolving architecture of the alveolar niche (92–94). As a result, macrophage states in ALI are best understood as dynamic tissue-adapted programs rather than fixed polarization categories.

High-dimensional and single-cell approaches have substantially advanced this view. Studies using CyTOF and single-cell transcriptomics in ARDS and experimental lung injury have identified multiple macrophage subsets with distinct molecular signatures, suggesting specialization in inflammatory activation, leukocyte recruitment, phagocytosis, tissue support, or resolution-associated functions. These findings reinforce the idea that macrophage heterogeneity is not simply noise within a single population, but an organized feature of pulmonary immune responses. Importantly, this heterogeneity may change over time. A macrophage program that is beneficial in the earliest phase of injury, when danger sensing and microbial containment are priorities, may become maladaptive if it persists into later stages when tissue clearance and repair should dominate. Conversely, premature induction of suppressive or reparative programs could potentially impair host defense when the initiating insult remains active.

This temporal dimension is critical. Macrophage plasticity in ALI should not be viewed merely as the capacity to adopt different phenotypes, but as the ability to transition through functionally appropriate states at the right time and in the right location. Disease severity may therefore reflect not only how strongly macrophages are activated, but whether their state transitions are properly synchronized with the needs of the tissue. If inflammatory programs are prolonged, if efferocytic and reparative programs fail to emerge, or if macrophages become trapped in dysfunctional intermediate states, the lung may remain locked in a cycle of damage amplification and incomplete recovery. In this sense, macrophage dysfunction in ALI is fundamentally a problem of mistimed or misdirected rewiring. This broader framework also allows a more refined interpretation of recent findings on trained immunity, efferocytosis, and reparative programming. For example, alveolar macrophages can acquire states that enhance injury resolution through improved clearance of apoptotic cells and activation of pathways such as KLF4-MERTK-mediated efferocytosis. These observations do not fit neatly into a simplistic anti-inflammatory label. Rather, they point to a specialized pro-resolving macrophage phenotype that emerges from context-specific rewiring of function. Similarly, macrophages that support epithelial proliferation or restrain inflammatory monocyte-derived macrophages through trophic mediators represent distinct reparative states that are better described in terms of functional modules than traditional polarization terms. Accordingly, moving beyond the M1/M2 dichotomy is not a matter of replacing old terminology with more complex labels for its own sake. It reflects a conceptual shift in how ALI pathogenesis is understood. Macrophages should be considered as dynamic components of an evolving tissue circuit, with their biological significance defined by timing, localization, lineage origin, and functional coupling to other cells (95–98). This perspective is especially important for therapeutic translation. Strategies that broadly suppress macrophage activation may be ineffective or even harmful if they inadvertently eliminate reparative or pro-resolving subsets. A more productive approach will require identifying which macrophage states drive inflammatory escalation, which support tissue recovery, and how transitions between these states can be guided in a phase-specific manner. **Table 1** summarizes representative original studies showing that macrophages in ALI are highly heterogeneous and dynamically reprogrammed across the phases of injury, resolution, and repair.

**Table 1:** Core original studies defining macrophage heterogeneity and plasticity in acute lung injury.

Study	PMID	Experimental setting	Main focus	Key findings	Relevance to this review
Frank et al., 2006 (70)	16877636	Ventilator-induced lung injury	Resident alveolar macrophages in barrier damage	Alveolar macrophages directly contributed to alveolar barrier dysfunction during ventilator-induced injury	Supports the concept that resident alveolar macrophages act as early sentinels and amplifiers of lung injury
Moon et al., 2015 (56)	26658190	Experimental ALI	Epithelial–macrophage communication via extracellular vesicles	Lung epithelial cell-derived extracellular vesicles activated macrophages and promoted inflammatory responses	Demonstrates that injured epithelial cells actively instruct macrophage activation in ALI
Soni et al., 2016 (80)	27287089	LPS-induced ALI	Macrophage-derived microvesicles	Alveolar macrophage-derived microvesicles mediated inflammatory lung injury	Supports the role of macrophages as active propagators of inflammatory signaling rather than passive responders
Morrell et al., 2018 (172)	29769438	Human ARDS samples	Alveolar macrophage heterogeneity	CyTOF identified distinct alveolar macrophage subtypes in ARDS	Provides key evidence that macrophage populations in lung injury are heterogeneous rather than uniform
Zhang et al., 2019 (103)	31331947	Experimental lung inflammation	Macrophage microvesicle cargo	Microvesicle-associated miR-223/142 regulated inflammatory signaling	Highlights the importance of macrophage-derived vesicular communication in shaping lung inflammation
Long et al., 2019	31801908	LPS-induced ALI	Macrophage signaling in resolution	MEK1 regulated macrophage inflammatory responses and influenced resolution of lung injury	Supports the idea that resolution is an active macrophage-governed process
Hung et al., 2019 (110)	30337651	Infectious and non-infectious lung injury	Macrophage-driven epithelial repair	Macrophages promoted epithelial proliferation through a Trefoil factor 2-dependent mechanism	Establishes macrophages as active coordinators of epithelial repair
Jiang et al., 2020 (117)	32554932	Human ARDS, single-cell analysis	Early monocyte programs	Single-cell RNA sequencing identified early monocyte-associated inflammatory signatures in ARDS	Supports the contribution of recruited monocyte-derived macrophage programs to inflammatory escalation
Mu et al., 2020 (111)	32793225	Experimental ALI	Epithelial instruction of alveolar macrophages	Alveolar epithelial cells promoted IGF-1 production by alveolar macrophages	Illustrates a reparative epithelial–macrophage communication axis
Gschwend et al., 2021 (37)	34431978	Lung developmental and homeostatic niche studies	AT2-derived GM-CSF and alveolar macrophages	Alveolar macrophages depended on epithelial GM-CSF for establishment and maintenance	Provides mechanistic support for the alveolar niche as a determinant of macrophage identity
Chakraborty et al., 2023 (66)	37615937	Experimental lung injury	Efferocytosis and trained reparative immunity	Trained alveolar macrophages enhanced injury resolution via KLF4–MERTK-dependent efferocytosis	Strong evidence for reparative macrophage rewiring beyond the M1/M2 framework
Hou et al., 2025 (112)	41207507	Experimental ALI	Resident–recruited macrophage interaction	Alveolar macrophage-derived TGF-β promoted recovery by restraining inflammatory monocyte-derived macrophages	Supports the idea that macrophage subset balance determines repair versus persistent injury

Several unresolved issues remain. First, it is still unclear in many ALI settings whether resident AM dysfunction primarily reflects phenotypic reprogramming, cell death, displacement by recruited monocyte-derived macrophages, or loss of epithelial niche support. Second, although single-cell studies have revealed substantial macrophage heterogeneity in ARDS and experimental lung injury, transcriptional states do not always map directly onto functional behavior. Lineage tracing, fate mapping, spatial transcriptomics, and time-resolved perturbation studies are needed to determine whether specific macrophage subsets actively drive injury, merely mark disease severity, or participate in repair. Third, the reparative potential of recruited monocyte-derived macrophages remains incompletely defined. These cells can amplify inflammation, but whether and when they can acquire AM-like, efferocytic, or epithelial-supportive functions is likely to depend on injury type, timing, and niche reconstruction. Addressing these questions will be essential for moving the field beyond descriptive macrophage classification toward a mechanistic understanding of macrophage state transitions in ALI.

## 4 Macrophages as orchestrators of resolution and tissue repair

### 4.1 Resolution is an active program rather than passive decline of inflammation

The resolution of acute lung injury is often described clinically as the disappearance of inflammatory abnormalities and the gradual restoration of pulmonary function. Mechanistically, however, resolution is not a passive waning of inflammatory activity once the initial insult subsides. It is an active, highly regulated biological program that requires dedicated cellular processes to terminate inflammation, remove cellular debris, restore epithelial integrity, and re-establish tissue homeostasis. Within this program, macrophages are central orchestrators. Their importance extends far beyond early inflammatory sensing; they are also indispensable for ensuring that acute immune activation transitions into controlled recovery rather than persistent tissue dysfunction. This distinction is critical because the failure of resolution is one of the key features separating self-limited lung injury from progressive ALI/ARDS with prolonged impairment. A lung that simply stops receiving inflammatory signals does not automatically return to health. Dying neutrophils and epithelial cells must be cleared, inflammatory mediators must be neutralized or counterbalanced, vascular and epithelial barriers must be rebuilt, and the local immune environment must be recalibrated from a state of danger toward one of restoration (99, 100). Macrophages are uniquely suited to coordinate these tasks because they combine sensing, effector, and regulatory functions within a single cellular system. When these functions are executed appropriately, inflammation resolves in an ordered manner. When they fail, the result is not only persistent inflammation but also disruption of the biological sequence required for repair. A central implication of this view is that macrophage activity during the resolution phase should not be interpreted as a mere attenuation of prior inflammatory behavior. Instead, resolution requires the emergence of specific macrophage programs that are qualitatively distinct from those operating during injury initiation. These include enhanced efferocytosis, altered cytokine production, restriction of further leukocyte recruitment, secretion of trophic and pro-repair mediators, and crosstalk with epithelial cells that promotes barrier restoration. Thus, the macrophage role in ALI is not biphasic in a simplistic sense of “first harmful, later beneficial,” but rather involves active functional rewiring in response to changing tissue demands.

Experimental evidence supports this interpretation. Perturbation of macrophage signaling pathways can impair the resolution of lung injury even when the initiating inflammatory trigger is no longer dominant, indicating that successful recovery depends on macrophage-intrinsic and macrophage-mediated programs rather than on spontaneous decay of inflammation alone. This helps explain why some cases of ALI remain unresolved despite apparent control of infection or initial injury. The persistence of tissue damage may reflect a failure to induce the macrophage-dependent machinery of repair, not just continued exposure to injurious stimuli. Viewing resolution as an active macrophage-governed program also reframes how disease progression should be interpreted. Inflammatory escalation and tissue recovery are not independent phases separated by a simple handoff. Instead, they overlap, and the seeds of failed resolution may already be planted early in injury if macrophage responses become excessively inflammatory, if resident populations are depleted, or if the tissue niche does not support later reparative reprogramming. In other words, the capacity to resolve inflammation is partly determined by how the early innate immune response is organized. This reinforces the broader theme of this review: ALI is best understood as a disorder of dynamic tissue immune circuits, in which the same cells that help initiate inflammation must later be redirected toward repair. For these reasons, the resolution phase deserves equal mechanistic attention as the injury phase itself. Understanding how macrophages terminate inflammation, transition into pro-resolving states, and interact with the recovering alveolar environment is essential for explaining the divergent outcomes of acute lung injury. It also has direct therapeutic relevance. Interventions that focus exclusively on suppressing early inflammation may miss a crucial opportunity to actively promote the cellular programs required for recovery. A circuit-based understanding of ALI therefore demands that macrophage-mediated resolution be recognized not as the endpoint of injury, but as a distinct and indispensable biological process in its own right.

### 4.2 Efferocytosis, reparative reprogramming, and macrophage functional transition

One of the defining features of macrophage-mediated resolution in acute lung injury is efferocytosis, the process by which macrophages recognize, engulf, and remove apoptotic cells (101). Although often described as a housekeeping function, efferocytosis is far more than passive debris disposal. In the injured lung, it represents a pivotal biological switch that links inflammatory termination to tissue recovery. By clearing apoptotic neutrophils, damaged epithelial cells, and other dying cellular elements, macrophages prevent secondary necrosis, limit the release of additional danger signals, and reduce the persistence of inflammatory stimuli within the alveolar space. In this way, efferocytosis directly restrains one of the major drivers of continued tissue injury.

Equally important, efferocytosis actively reprograms macrophage behavior. The engulfment of apoptotic material does not merely eliminate harmful debris; it reshapes macrophage transcriptional, metabolic, and secretory programs in ways that favor resolution (102). Macrophages that efficiently perform efferocytosis tend to reduce pro-inflammatory signaling, enhance anti-inflammatory and pro-resolving mediator production, and adopt functions that support epithelial repair and restoration of alveolar homeostasis. This transition is especially relevant in ALI, where the burden of dying cells is high and the need for coordinated inflammatory shutdown is immediate. If efferocytosis is inefficient, delayed, or overwhelmed, the inflammatory environment remains active, neutrophil-rich injury persists, and the tissue fails to transition into a reparative state.

Recent work has further expanded this concept by showing that efferocytosis is embedded within broader macrophage programs of functional rewiring (103). In particular, pathways related to KLF4-MERTK-mediated efferocytosis and trained immunity-associated resolution suggest that macrophages can acquire specialized states that are optimized for injury clearance and repair rather than merely displaying a generic anti-inflammatory phenotype (66). These findings are important because they highlight that macrophage transition during recovery is not simply a relaxation of inflammatory tone, but the active acquisition of new functional modules suited to the needs of the injured tissue. Such modules include enhanced apoptotic cell clearance, altered responsiveness to local cytokines, improved support of epithelial regeneration, and increased capacity to stabilize the recovering microenvironment (104–106). This perspective also helps explain why macrophage plasticity is so consequential in ALI. Resolution depends not only on the presence of macrophages, but on whether they can successfully traverse the transition from inflammatory amplification to reparative coordination. This transition is shaped by multiple cues, including the burden of apoptotic cells, epithelial-derived growth factors, tissue metabolic conditions, and the status of resident versus recruited macrophage populations. A macrophage compartment that remains locked in inflammatory activation, or one that is unable to perform efficient efferocytosis, becomes an obstacle to recovery rather than an engine of repair. Thus, failed macrophage transition can be understood as one of the central mechanisms linking acute tissue damage to prolonged pulmonary dysfunction. The concept of reparative reprogramming also broadens the therapeutic horizon. It suggests that successful intervention in ALI may require more than suppressing inflammatory cytokine production. Promoting macrophage efferocytosis, enhancing pro-resolving state transitions, and restoring the tissue signals that support reparative programming may be equally important, particularly once injury has been established. This is especially relevant because the same macrophage population that contributed to early inflammatory escalation may later need to be preserved and redirected rather than simply eliminated. In this sense, macrophage functional transition is not a peripheral feature of recovery, but a central determinant of whether the injured lung remains trapped in inflammation or moves toward tissue restoration.

### 4.3 Macrophage–epithelial repair circuits

The restoration of alveolar integrity after acute lung injury requires close cooperation between immune and structural cells, and among these interactions, macrophage–epithelial communication is especially important (107–109). The alveolar epithelium is both a major target of injury and the essential substrate for subsequent repair. Because gas exchange depends on the rapid recovery of epithelial continuity and function, any successful resolution program must include mechanisms that support epithelial survival, proliferation, differentiation, and barrier re-establishment. Macrophages are now recognized as central contributors to this process. Beyond clearing debris and restraining inflammation, they actively shape the epithelial repair response through trophic signaling and niche reconstruction. This reparative role is conceptually important because it challenges older models in which immune cells and tissue regeneration were treated as largely separate domains. In ALI, macrophages are not merely tasked with “getting out of the way” once inflammation declines. Rather, they help create the conditions under which epithelial regeneration can occur. Experimental studies have shown that macrophages can promote epithelial proliferation through mediators such as trefoil factor 2, demonstrating that they participate directly in the restoration of the alveolar lining rather than merely influencing it indirectly (110). Likewise, macrophage-derived factors such as IGF-1 and TGF-β have emerged as important components of recovery-associated signaling, further supporting the view that macrophages function as local coordinators of regeneration (111, 112). These repair circuits are bidirectional. Injured epithelial cells do not simply receive macrophage-derived support; they also instruct macrophage behavior through growth factors, cytokines, extracellular vesicles, and niche-dependent signals. As injury resolves, epithelial restoration and macrophage reprogramming become mutually reinforcing processes. Epithelial signals can help stabilize reparative macrophage states, while macrophages can promote epithelial proliferation and constrain inflammatory monocyte-derived populations that would otherwise continue to damage the tissue. This reciprocity is particularly evident in the balance between resident alveolar macrophages and recruited monocyte-derived macrophages. Resident macrophages, when appropriately maintained or restored, may suppress excessive inflammatory dominance and help establish a tissue environment more conducive to epithelial recovery.

Importantly, these macrophage–epithelial circuits are not uniformly protective in all settings. Their success depends on timing, signal intensity, and the extent of niche disruption. Severe epithelial loss, persistent alveolar flooding, oxidative stress, or continued influx of inflammatory myeloid cells may all interfere with the emergence of productive repair signaling. Under such conditions, macrophages may fail to fully transition into trophic states or may provide insufficient support for epithelial regeneration. Alternatively, epithelial cells may no longer be capable of appropriately instructing or responding to reparative immune cues. In either case, the breakdown of macrophage–epithelial reciprocity contributes to ongoing barrier dysfunction and delayed return to homeostasis (113–116). This framework highlights an important principle: repair after ALI is not solely a property of epithelial progenitor capacity, nor is it solely determined by the strength of inflammatory suppression. It is an emergent property of coordinated cell–cell communication within the recovering alveolar niche. Macrophages, by virtue of their ability to sense damage, clear debris, interpret tissue signals, and secrete regenerative mediators, are uniquely positioned to bridge these domains. Therefore, understanding macrophage–epithelial repair circuits is essential for explaining why some injured lungs regenerate effectively while others remain in a state of incomplete recovery. It also suggests that therapeutic strategies aimed at restoring alveolar function should prioritize not only epithelial protection but also the immune signals that make epithelial repair biologically possible.

### 4.4 Why repair fails in severe or persistent ALI

A defining feature of severe or non-resolving acute lung injury is that the tissue does not simply remain inflamed; it fails to complete the transition from injury control to structural recovery. This distinction is important. Repair failure is not merely the prolonged presence of inflammation, nor is it synonymous with the severity of the initial insult. Rather, it reflects a breakdown in the coordinated cellular programs required for clearance, regeneration, and re-establishment of tissue equilibrium. In this context, the concept of repair failure helps explain why some lungs remain functionally compromised long after the initiating trigger has diminished or been removed.

One major contributor to repair failure is the persistence of inflammatory skewing within the macrophage compartment. When recruited monocyte-derived macrophages remain dominant, when resident alveolar macrophages are depleted or functionally impaired, or when macrophages fail to transition into pro-resolving states, the alveolar environment remains biased toward ongoing injury rather than recovery. Under these conditions, cytokine production, endothelial dysfunction, and epithelial stress continue to reinforce one another, preventing the emergence of a stable reparative niche (117–119). The problem is therefore not only that inflammation is “too strong,” but that the cellular balance required to terminate inflammation and support tissue restoration is not successfully reconstituted. Defective efferocytosis is another key mechanism. If apoptotic neutrophils and damaged parenchymal cells are not efficiently removed, the alveolar space remains rich in pro-inflammatory and danger-associated signals. This perpetuates innate immune activation, delays macrophage reprogramming, and creates a tissue environment in which epithelial regeneration becomes progressively more difficult. In this sense, failed clearance acts as a bottleneck linking unresolved inflammation to failed repair. It prevents the biological shift from destruction to reconstruction and may also promote secondary tissue injury through the accumulation of necrotic debris and amplifying inflammatory mediators.

**Figure 2:**
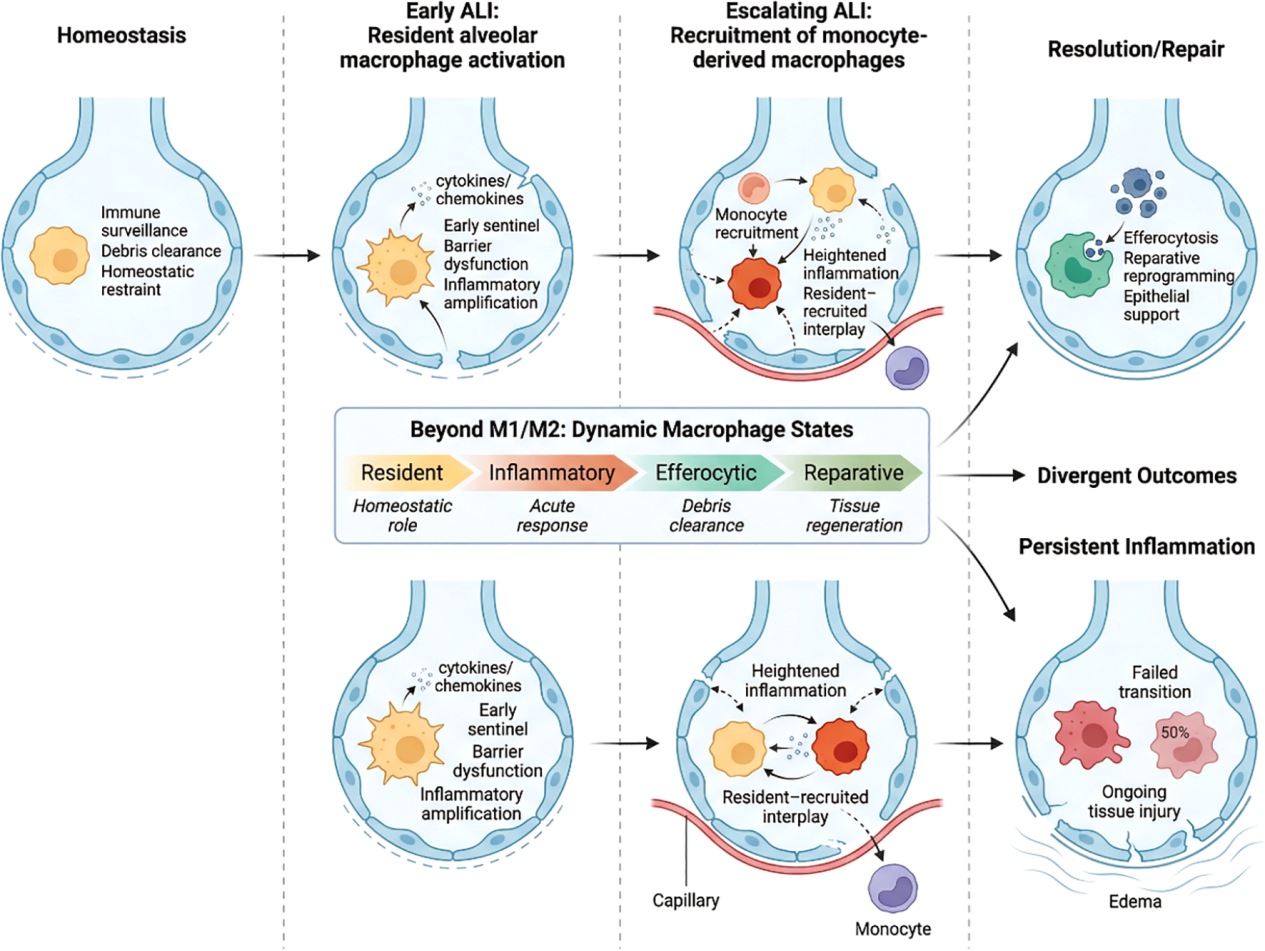
Dynamic macrophage state transitions in acute lung injury. Resident alveolar macrophages act as early sentinels that initiate inflammatory responses after epithelial injury, whereas recruited monocyte-derived macrophages amplify tissue damage during escalating ALI. Rather than fitting a simple M1/M2 model, macrophages undergo context- and time-dependent functional rewiring, ultimately shifting toward either efferocytic, reparative states that support resolution or persistent inflammatory states associated with failed repair.

Repair failure can also arise from disruption of the structural niche itself. Because macrophage reparative behavior depends on tissue-derived cues, severe epithelial damage has consequences that extend beyond barrier dysfunction. Loss of epithelial integrity means loss of local signals that normally support resident macrophage identity, regulate immune restraint, and instruct regenerative programs. If this niche is not restored, even macrophages capable of reparative function may be unable to sustain the appropriate phenotype. Similarly, when epithelial cells remain metabolically compromised or functionally stressed, their capacity to respond to macrophage-derived trophic signals is diminished. Thus, failed repair is often best understood not as the defect of one cellular population alone, but as the collapse of reciprocal support between immune and structural compartments. These mechanisms also help explain why the outcomes of ALI are so heterogeneous. Patients exposed to similar inflammatory triggers may diverge sharply in recovery because the success of repair depends on variables not fully captured by traditional measures of inflammatory burden. The preservation of resident macrophage niches, the efficiency of apoptotic cell clearance, the plasticity of recruited macrophages, the integrity of epithelial signaling, and the timing of immune state transitions may all influence whether tissue repair proceeds effectively. A circuit-based model therefore provides a more nuanced explanation for clinical variability than one based solely on cytokine magnitude or neutrophil abundance. From a translational perspective, this view carries important implications. It suggests that therapies aimed only at blunting early inflammation may be insufficient once the lung has entered a state of failed repair. In such settings, recovery may require active restoration of macrophage efferocytosis, re-establishment of epithelial–immune communication, suppression of persistent inflammatory monocyte-derived programs, and support for tissue-regenerative signaling. Repair failure is therefore not merely a late consequence of ALI, but a distinct pathogenic state that deserves direct mechanistic and therapeutic attention. Understanding how and why the lung becomes trapped in this state is central to any effort to improve long-term outcomes after acute pulmonary injury. **Figure 2** illustrates that macrophage responses in ALI are dynamically shaped by lineage origin, tissue context, and timing rather than by a static M1/M2 polarization framework.

## 5 ILCs in ALI: tissue alarm sensing, repair support, and context-dependent contribution

Although this review emphasizes ILC2-associated repair programs, the contribution of ILCs to ALI should be interpreted within the broader immune network rather than in isolation. In most forms of ALI/ARDS, macrophages, recruited monocytes, neutrophils, endothelial cells, epithelial cells, dendritic cells, T cells, and regulatory T cells all participate in disease initiation, inflammatory amplification, resolution, and repair (120, 121). Neutrophils are major acute effector cells that promote microbial defense but also contribute to epithelial–endothelial barrier injury through proteases, reactive oxygen species, and neutrophil extracellular traps (122, 123). Macrophages and monocyte-derived macrophages coordinate danger sensing, cytokine production, efferocytosis, and tissue repair (124, 125). T cells and regulatory T cells may further influence inflammatory persistence, immune suppression, and resolution (121, 126). Compared with these populations, ILCs are generally less abundant but may exert disproportionate effects in specific tissue niches because they respond rapidly to epithelial-derived alarmins and produce mediators such as amphiregulin, IL-5, IL-9, and IL-13 (127–129). Therefore, ILCs should be viewed as context-dependent modulators of epithelial–immune communication and repair support, rather than as dominant drivers of ALI pathogenesis.

### 5.1 ILCs as tissue-resident sensors and effectors in injured lungs

Innate lymphoid cells have emerged as important regulators of tissue immunity at barrier surfaces, and the lung is one of their most functionally significant sites (130–132). Unlike adaptive lymphocytes, ILCs do not rely on antigen-specific receptors to initiate responses. Instead, they are poised to respond rapidly to local tissue-derived signals, placing them among the earliest immunological interpreters of epithelial stress and barrier disruption. This property makes them particularly relevant to acute lung injury, where the earliest determinants of outcome are often encoded not by antigen-specific immunity, but by how the tissue senses and responds to danger. Among pulmonary ILC populations, ILC2s have attracted the greatest attention because of their established roles in mucosal homeostasis, epithelial support, and type 2 immune responses. However, their significance in acute injury extends beyond classical allergic paradigms. In the context of tissue damage, ILC2s can function as sensors of epithelial alarm signals and as downstream effectors that help coordinate protective adaptation (133–135). This is especially important in the lung, where epithelial integrity is tightly linked to organ function and where timely restoration of the barrier is essential for survival. Accordingly, ILC2s should not be viewed solely as cytokine-producing lymphoid cells, but as tissue-resident participants in the early orchestration of injury responses.

This perspective reframes the biology of ILCs in ALI. Rather than entering the picture only after inflammation is established, ILCs may help determine the trajectory of injury from an early stage by translating epithelial distress into broader tissue-level programs. Because they are responsive to local cytokines and alarmins released by damaged epithelium, ILCs are well positioned to integrate signals of tissue disruption with signals of repair demand. Depending on the surrounding context, this may result in the production of mediators that enhance barrier support, promote epithelial survival, or shape the activation states of other innate immune cells. Thus, ILCs occupy a strategic position within the pulmonary immune network, linking structural damage to adaptive tissue responses. At the same time, the role of ILCs in acute lung injury is not uniformly protective. Their activation is highly context dependent, influenced by the nature of the insult, the intensity of epithelial injury, the presence or absence of infection, and the broader inflammatory milieu (136, 137). In some settings, ILC-associated programs may support restoration of homeostasis; in others, they may contribute to maladaptive remodeling or reinforce inappropriate immune bias. This duality is important because it prevents overly simplistic interpretations of ILCs as either beneficial repair cells or secondary inflammatory amplifiers. As with macrophages, their biological significance lies in timing, tissue context, and the balance of signals they receive and transmit. Recognizing ILCs as tissue-resident sensors and effectors also strengthens the broader argument of this review: ALI is best understood as a disorder of innate immune circuits rather than a purely myeloid or cytokine-centered process. The lung contains multiple resident immune populations capable of rapidly interpreting structural damage, and ILCs are among the most important of these. Their ability to respond early, communicate with epithelial cells, and influence the behavior of macrophages and other immune populations makes them key participants in the transition from injury sensing to repair control. For this reason, any integrated model of acute lung injury that aims to explain both inflammatory amplification and recovery must account for the contribution of ILCs within the tissue-level immune network.

### 5.2 ILC2-dependent epithelial maintenance and lung homeostasis

A central reason for focusing on ILC2s in acute lung injury is that these cells are increasingly recognized as regulators of epithelial maintenance rather than merely participants in inflammatory signaling. The pulmonary epithelium is not only the first structure damaged in many forms of ALI, but also the key determinant of whether the lung can regain functional integrity after the inflammatory phase subsides. Because ILC2s are highly responsive to epithelial-derived alarm signals and are capable of producing mediators linked to tissue support, they occupy a strategic position at the interface between damage detection and barrier restoration (138, 139).

This reparative role is conceptually important because it broadens the conventional view of ILC2 biology. In many discussions, ILC2s are framed mainly through the lens of type 2 cytokines and allergic airway disease. While that framework remains relevant, it does not fully capture their role in injured tissue settings. In the lung, ILC2s can promote epithelial recovery through the production of amphiregulin and other mediators that support epithelial survival, proliferation, and restoration of tissue architecture (127, 140). These functions suggest that ILC2s are not simply inflammatory amplifiers but part of a local homeostatic system that helps the tissue adapt to and recover from injury.

The significance of this function becomes especially clear when considering the temporal demands of ALI. Once the initial insult has damaged the alveolar barrier, the tissue requires not only suppression of excessive inflammation but also the rapid re-establishment of epithelial continuity. ILC2-associated programs are well suited to this task because they can be activated quickly by local tissue signals and can deliver repair-supportive outputs without the delay required for antigen-specific adaptive responses (141). This rapidity may be particularly important in the lung, where prolonged epithelial vulnerability can perpetuate edema, impair gas exchange, and expose the tissue to ongoing inflammatory and mechanical stress.

ILC2-dependent tissue maintenance is also notable because it reflects a broader principle of barrier immunity: the same cells that respond to danger are often also involved in reconstructing local equilibrium. In this sense, ILC2s resemble reparative macrophages in that their role cannot be reduced to either defense or repair alone. Rather, they participate in a continuum of responses that begins with tissue alarm sensing and extends into homeostatic restoration (142). Whether this program becomes successful in ALI depends on the surrounding microenvironment, including the intensity of epithelial damage, the availability of supportive niche signals, and the influence of other immune cell populations. This framework also helps explain why impairment of ILC2-associated repair pathways may contribute to failed recovery. If ILC2 activation is insufficient, mistimed, or distorted by an overwhelmingly inflammatory milieu, epithelial support may become inadequate even if some degree of inflammation control is achieved. Under such conditions, the lung may remain trapped in a state of structural vulnerability, where incomplete barrier restoration feeds back into continued immune activation. Thus, defective ILC2-mediated epithelial maintenance can be understood not merely as the absence of a beneficial pathway, but as a mechanistic contributor to the transition from acute injury to persistent tissue dysfunction. For these reasons, ILC2s should be considered important components of the endogenous repair machinery in ALI. Their ability to couple epithelial alarm sensing with tissue-protective output makes them highly relevant to the question of why some injured lungs return to homeostasis while others do not. Integrating ILC2 biology into models of ALI therefore provides a more complete understanding of recovery, emphasizing that successful resolution depends not only on shutting down inflammation but also on actively supporting epithelial regeneration through tissue-resident innate immune programs.

The relative contribution of ILC2s is also likely to vary according to the stage and etiology of ALI. During the early exudative phase, neutrophils, resident alveolar macrophages, recruited monocytes, endothelial activation, and epithelial barrier disruption are usually the dominant drivers of inflammatory injury. In contrast, ILC2-associated programs may become more relevant during the transition from inflammation to resolution, when epithelial survival, barrier restoration, and tissue remodeling become critical determinants of outcome. Even in this phase, ILC2s are unlikely to act alone; their effects depend on the surrounding macrophage state, epithelial niche integrity, stromal responses, and the persistence or clearance of neutrophil-rich inflammation. Thus, the importance of ILC2s in ALI is best understood as phase-dependent and niche-dependent, with their major contribution lying in the modulation of repair-associated communication rather than in replacing the established roles of myeloid and neutrophil pathways.

### 5.3 Environmental cues and temporal control of ILC responses

The contribution of ILCs to acute lung injury cannot be understood without considering the environmental cues that shape their activation and function. Unlike adaptive lymphocytes, which depend on antigen-specific recognition and clonal expansion, ILCs are deeply embedded within the local tissue environment and are instructed primarily by contextual signals (143). In the lung, these signals include epithelial-derived alarmins, inflammatory cytokines, stromal inputs, metabolic conditions, and the degree of structural disruption within the alveolar niche. As a result, ILC behavior is highly sensitive to both the nature of the insult and the evolving stage of the injury response. This contextual dependence is particularly important in ALI, where the pulmonary environment changes rapidly over time. In the early phase of injury, epithelial damage and barrier disruption create a setting rich in stress signals that can activate ILCs as part of an immediate tissue response. At this stage, ILC activation may serve adaptive purposes, including support of barrier protection and the initiation of reparative signaling. However, as injury progresses, the composition of the tissue environment changes. Persistent inflammatory mediators, metabolic stress, edema, altered oxygen availability, and changes in immune cell composition may all reshape ILC responses (144–146). Thus, the biological meaning of ILC activation in ALI is not fixed; it evolves along with the tissue microenvironment. This temporal dimension has major implications for interpretation. An ILC program that is beneficial early in injury may become insufficient, maladaptive, or even pathogenic if it persists under the wrong conditions or fails to transition appropriately. Conversely, delayed or inadequate activation of protective ILC pathways may contribute to ineffective tissue restoration. In this respect, ILC biology in ALI mirrors the broader principle already discussed for macrophages: the critical issue is not simply whether a cell type is activated, but whether it is activated in the right way, at the right time, and within the right tissue context. Disease progression may therefore reflect a loss of temporal coordination as much as a loss of immune restraint. Environmental control of ILC function also reinforces the idea that these cells participate in tissue-level circuits rather than acting as isolated effectors. Epithelial cells are central to this process because they provide many of the earliest and most influential signals that shape ILC activity. At the same time, macrophages and other innate immune cells contribute additional cues that may bias ILCs toward protective, inflammatory, or remodeling-associated programs. The pulmonary environment therefore does not simply trigger ILC responses; it continually modulates them, integrating structural damage with immune adaptation. This makes ILCs particularly relevant to the concept of tissue-driven inflammation that underlies the framework of this review. Another important implication is that the same ILC subset may play distinct roles across different forms of lung disease or even across different phases of the same disease. Findings from viral lung injury, epithelial injury models, and chronic airway inflammation collectively suggest that ILCs possess substantial functional flexibility (147–149). While this flexibility is essential for tissue adaptation, it also complicates therapeutic targeting. Broad stimulation or suppression of ILC pathways may have very different consequences depending on when during the disease course intervention occurs and what state the pulmonary niche is in at that moment. Appreciating this complexity is necessary if ILC-directed therapies are to be developed rationally for ALI.

**Figure 3:**
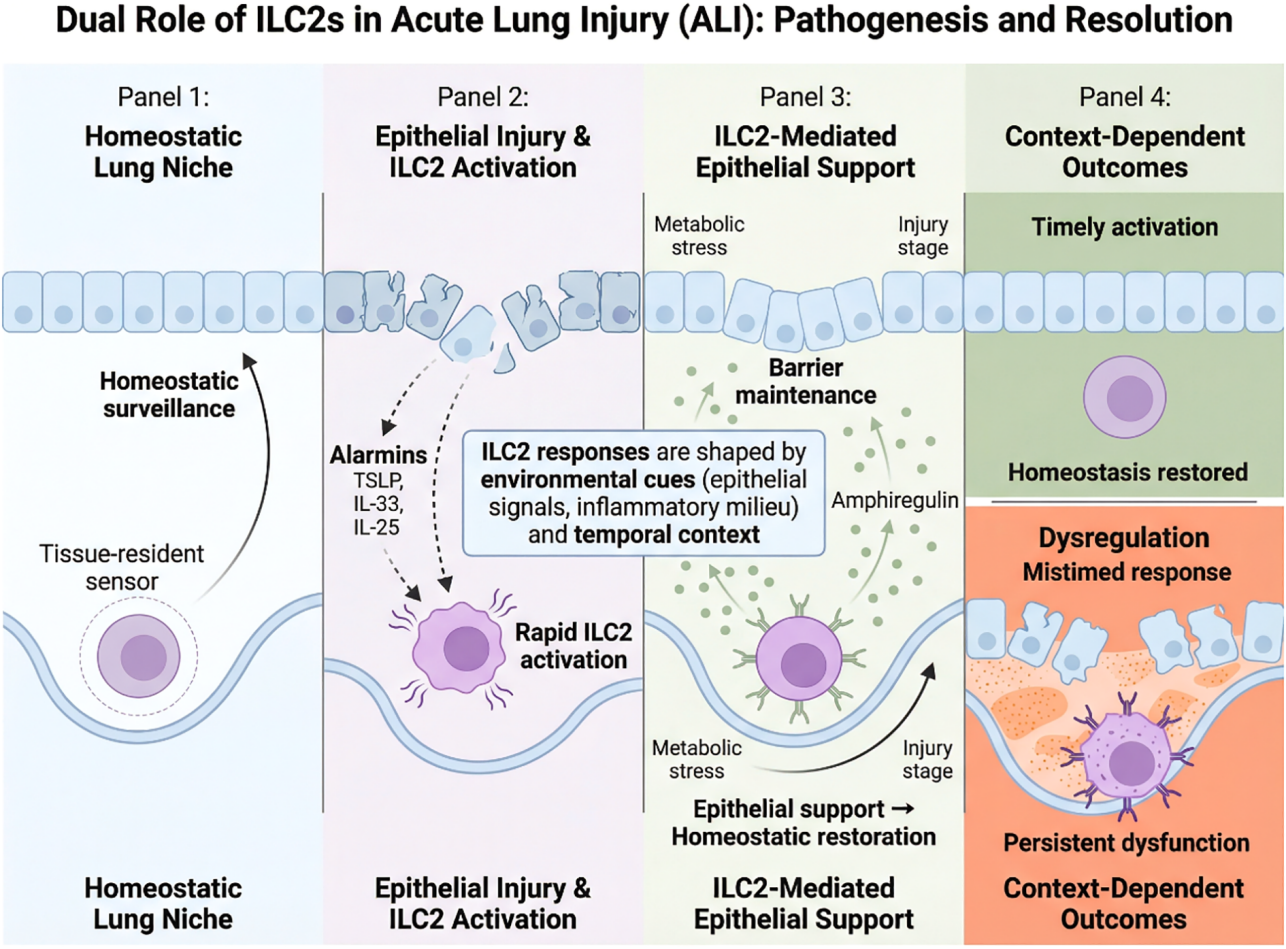
From epithelial alarm sensing to repair control: ILC2 responses in acute lung injury. In ALI, epithelial injury releases local alarm signals that rapidly activate tissue-resident ILC2s. These cells support epithelial maintenance, barrier integrity, and homeostatic restoration through context-dependent repair programs. However, because ILC2 activity is shaped by environmental cues and injury stage, mistimed or dysregulated responses may fail to restore tissue integrity and instead contribute to persistent dysfunction.

Taken together, these observations support a dynamic model in which ILC responses are governed by environmental instruction and temporal control. Their relevance to ALI lies not only in what they are capable of producing, but in how their activity is sculpted by injury stage, tissue context, and intercellular communication. This perspective also provides a bridge to the next major concept of this review: because ILCs are so tightly conditioned by the tissue environment and by other innate immune cells, their function in ALI is inseparable from their crosstalk with macrophages. Understanding how these two systems interact is therefore essential for explaining the balance between inflammatory amplification and successful repair. **Table 2** highlights key original studies indicating that ILCs, especially ILC2s, act as tissue-responsive regulators of epithelial maintenance, lung homeostasis, and macrophage-associated immune adaptation. **Figure 3** illustrates that ILC2s function as tissue-resident interpreters of epithelial stress, linking local alarm sensing to epithelial support and lung homeostasis in ALI.

**Table 2:** Key original studies supporting ILC-mediated tissue adaptation, epithelial maintenance, and macrophage–ILC crosstalk in lung injury.

Study	PMID	Experimental setting	Main focus	Key findings	Relevance to this review
Monticelli et al., 2011 (127)	21946417	Influenza-induced lung injury	ILCs in tissue homeostasis	Innate lymphoid cells promoted lung tissue homeostasis after viral injury	Landmark study supporting ILC-dependent lung repair and homeostatic restoration
Mohapatra et al., 2016 (140)	26129648	Lung homeostasis model	ILC2–epithelial maintenance axis	ILC2s used the IRF4–IL-9 module to support epithelial maintenance and lung homeostasis	Demonstrates that ILC2s actively sustain epithelial integrity
Puttur et al., 2019 (143)	31175176	Mouse and human lung tissue	Environmental control of ILC2 dynamics	Pulmonary environmental cues shaped ILC2 localization and functional dynamics	Supports the concept that ILC responses are tissue-instructed and context dependent
Kim et al., 2019 (157)	30414858	Asthmatic airway inflammation	ILC–macrophage crosstalk	Innate lymphoid cells coordinated polarization of lung macrophages	Provides direct evidence that ILCs can shape macrophage functional bias in the lung
Wu et al., 2022 (141)	35030033	Acute respiratory viral infection	ILC2-mediated tissue protection	BATF promoted ILC2-dependent lung tissue protection	Supports the protective and reparative role of ILC2-associated programs in acute lung injury settings
Jung et al., 2024 (142)	39423283	Epithelial injury model	ILC2-dependent restoration of homeostasis	An ILC2–chitinase circuit restored lung homeostasis after epithelial injury	Strong recent evidence linking ILC2 activity to epithelial recovery and repair control

## 6 Macrophage–ILC–epithelial crosstalk as an emerging framework for inflammatory amplification and repair failure

Although macrophage–ILC interactions provide an attractive framework for understanding the balance between inflammatory amplification and tissue repair, the strength of evidence differs across experimental contexts. Several findings are well established: macrophage populations are dynamically remodeled during ALI/ARDS; epithelial injury generates alarmin and danger signals that shape local innate immunity; and ILC2s can respond to epithelial-derived cytokines and contribute to barrier protection and tissue repair in the lung. However, direct evidence that macrophage–ILC circuits causally determine repair failure in human ALI/ARDS remains limited. Therefore, in this section, we distinguish established observations from emerging hypotheses and interpret macrophage–ILC crosstalk as a developing conceptual model rather than a fully proven mechanism in human disease.

### 6.1 Conceptualizing macrophage–ILC circuits in ALI

A central argument of this review is that macrophages and ILCs should not be viewed as parallel but separate contributors to acute lung injury. Instead, they are best understood as components of an integrated innate immune circuit in which each population shapes the activation state, functional output, and tissue effects of the other. This perspective is important because it moves beyond cell-by-cell descriptions of ALI pathogenesis and toward a network-based model capable of explaining why the injured lung may follow divergent trajectories of inflammation, resolution, or failed repair.

Macrophages and ILCs are particularly well suited to form such circuits because both are tissue-embedded, highly responsive to local cues, and capable of rapidly influencing the surrounding microenvironment (150–153). Macrophages interpret signals of cellular damage, infection, and structural disruption, while ILCs respond quickly to epithelial alarm programs and help translate tissue stress into barrier-adaptive responses. These two systems operate within the same pulmonary niche and are exposed to many of the same environmental inputs, including cytokines, growth factors, extracellular vesicles, metabolic stress signals, and structural cell-derived mediators. It would therefore be biologically implausible for them to act independently. The more coherent interpretation is that they engage in reciprocal communication that helps define the overall direction of tissue immunity. Conceptualizing macrophage–ILC crosstalk as a circuit also helps explain why acute lung injury cannot be fully understood through the behavior of any single cell type alone. For example, a pro-inflammatory macrophage response may become especially damaging if it suppresses or distorts ILC-associated repair programs. Conversely, ILC activation may have different consequences depending on whether it occurs in the presence of inflammatory monocyte-derived macrophages, resident reparative alveolar macrophages, or a severely damaged epithelial niche (154–156). Thus, the effect of one population depends on the state of the others. This interdependence is a defining feature of circuit biology and is central to understanding how local immune responses scale into either restoration or persistent dysfunction.

Within this framework, macrophage–ILC circuits can be thought of as regulators of threshold, directionality, and timing. They help determine how strongly inflammatory responses are amplified, whether reparative programs are initiated early enough, and whether tissue recovery can outcompete ongoing damage. In the healthy or successfully recovering lung, these interactions may reinforce homeostatic restoration by coupling damage sensing with epithelial support and inflammatory control. In severe or persistent injury, however, the same network may become dysregulated, leading to exaggerated inflammation, mistimed repair signaling, or collapse of the coordination required for tissue recovery. This circuit-centered view is also valuable because it aligns closely with current advances in tissue immunology. Modern studies increasingly emphasize that disease is driven not only by cell abundance or cytokine levels, but by communication hubs and state transitions within local cell networks. Applying this principle to ALI suggests that macrophage–ILC interactions may represent one of the key communication modules that connect tissue alarm sensing to outcome. Rather than asking only how macrophages contribute to injury or how ILCs contribute to repair, it becomes more informative to ask how their reciprocal regulation shapes the balance between inflammatory amplification and reparative control. For these reasons, macrophage–ILC crosstalk should be considered an organizing principle rather than a secondary detail in ALI biology. It provides a unifying lens through which seemingly disparate findings on epithelial injury, macrophage plasticity, ILC2-mediated tissue support, and repair failure can be brought into a single mechanistic framework. This framework is especially useful for explaining why the injured lung is not simply inflamed, but dynamically poised between recovery and collapse depending on how its innate immune circuits are wired and rewired over time.

### 6.2 ILC-driven macrophage polarization and functional bias

One of the clearest ways in which macrophage–ILC crosstalk may influence the course of acute lung injury is through the ability of ILCs to shape macrophage functional bias. Although this concept has been more directly demonstrated in some airway inflammatory settings than in classical ALI models, it provides an important mechanistic foundation for understanding how lymphoid-like innate cells can regulate myeloid cell behavior within the lung (157). Rather than acting solely as independent producers of cytokines, ILCs may help determine whether macrophages remain predominantly inflammatory, acquire tissue-supportive properties, or become locked in maladaptive intermediate states. This possibility is highly relevant to ALI because macrophage state transitions are central to both injury progression and recovery. If ILC-derived signals favor macrophage programs associated with inflammatory restraint, epithelial support, or repair coordination, then ILC activity may indirectly promote resolution even without directly acting on structural cells. Conversely, if the surrounding context biases ILC responses in ways that reinforce inappropriate macrophage activation, the net effect may be persistent tissue injury (158–160). Thus, the significance of ILCs in ALI may lie partly in their capacity to modulate the macrophage compartment rather than merely contributing their own independent outputs.

The idea of ILC-driven macrophage polarization also reinforces the inadequacy of viewing pulmonary innate immunity in isolated compartments. Macrophage phenotype is not determined solely by pathogen sensing or direct epithelial instruction. It is also shaped by the broader cytokine and growth factor environment, of which ILCs may be an important source. In this sense, ILCs can be understood as contextual regulators of macrophage plasticity. They may influence how macrophages interpret tissue damage, how rapidly they transition toward reparative states, and how effectively they participate in epithelial restoration (161, 162). This is particularly important in a disease like ALI, where timing and coordination matter as much as the magnitude of immune activation. Another important implication is that ILC–macrophage communication may amplify either beneficial or harmful trajectories depending on the state of the tissue niche. In a recovering lung, ILC-mediated biasing of macrophages toward homeostatic or reparative programs could help consolidate resolution. In a severely inflamed or structurally compromised lung, however, the same communication network may fail to produce a restorative outcome because macrophages are simultaneously exposed to dominant pro-inflammatory signals from recruited monocytes, damaged epithelium, or persistent danger-associated cues. Thus, ILC influence on macrophages should not be understood as deterministic, but as one component of a broader decision-making network embedded in the injured tissue. This concept also provides a useful way to interpret why direct evidence for macrophage–ILC crosstalk in ALI remains relatively limited while the framework remains biologically compelling. The absence of extensive direct ALI-specific literature does not undermine the relevance of the interaction; rather, it reflects the fact that the field has only recently begun to appreciate lung injury as a problem of coordinated tissue circuits. Findings from neighboring pulmonary settings, particularly those demonstrating ILC-dependent regulation of macrophage polarization, suggest mechanisms that are highly plausible in ALI and deserve to be tested more directly. Including this perspective in the review therefore adds conceptual depth rather than speculative excess.

Overall, ILC-driven macrophage bias offers an important explanation for how pulmonary innate immunity becomes coordinated across distinct cell lineages. It suggests that successful recovery from ALI may require not only the emergence of reparative macrophages and protective ILCs as separate populations, but the establishment of reciprocal signaling that allows each to reinforce the beneficial state of the other. When this reciprocity is absent, incomplete, or overwhelmed by inflammatory stress, the circuit may fail to support resolution and instead contribute to persistent tissue dysfunction.

### 6.3 Epithelial cells as intermediaries in macrophage–ILC communication

Although macrophages and ILCs can influence one another through immune-derived mediators, much of their relationship in the lung is likely organized through epithelial cells. The epithelium is the central structural interface of the alveolar niche, the earliest site of injury in many forms of ALI, and one of the most important sources of local signals that instruct innate immune behavior. For this reason, macrophage–ILC communication in the injured lung is often best understood not as a simple two-cell interaction, but as a triadic network in which epithelial cells act as both initiators and integrators of immune crosstalk. This intermediary role is mechanistically plausible for several reasons. First, epithelial cells provide many of the alarm signals that rapidly activate ILCs in response to tissue damage. Second, epithelial cells also shape macrophage behavior by delivering growth factors, extracellular vesicles, and niche-maintaining signals that influence macrophage identity, activation threshold, and reparative potential (163–165). Third, both macrophages and ILCs feed back onto the epithelium by altering inflammatory tone, epithelial survival, proliferative capacity, and the restoration of barrier integrity. The result is a highly interdependent system in which epithelial state helps determine the direction of macrophage–ILC interactions, while immune cell outputs in turn influence whether epithelial recovery succeeds. This triadic perspective is especially useful for explaining why similar immune populations may behave differently across distinct forms or phases of ALI. Macrophages and ILCs do not communicate in a vacuum; they do so in an epithelial landscape that may range from mildly stressed to catastrophically disrupted. When epithelial cells remain sufficiently functional to provide organized alarm and niche signals, macrophage–ILC interactions may promote adaptive tissue responses. When epithelial injury becomes severe, however, the signaling architecture itself begins to collapse. Under such conditions, macrophages may lose key homeostatic instructions, ILCs may receive distorted or inadequate activation cues, and reciprocal immune support for epithelial repair may become progressively ineffective. Thus, the quality of the epithelial intermediary determines not only whether immune cells are activated, but whether their crosstalk remains constructive.

Viewing epithelial cells as intermediaries also helps unify otherwise separate strands of the literature. Studies on epithelial extracellular vesicles, AT2-derived niche signals, ILC2 activation by tissue-derived mediators, and macrophage-driven epithelial repair can all be interpreted as different aspects of the same overarching network (166–168). This interpretation is particularly valuable for a review focused on innate immune circuits, because it emphasizes that the fundamental pathogenic unit in ALI is not a cell type, but a communication system. The key question is not simply whether macrophages or ILCs are present, but whether epithelial-centered signaling networks are sufficient to coordinate them toward restoration. This framework has important consequences for understanding repair failure. If epithelial cells are too damaged to provide the cues necessary for productive immune crosstalk, then even macrophages and ILCs with intact reparative potential may fail to restore the tissue. Conversely, if epithelial alarm programs remain excessive or chronically dysregulated, they may continuously drive inflammatory macrophage activation and maladaptive ILC responses. In either case, the collapse of epithelial intermediary function contributes directly to the failure of innate immune circuits to transition from damage response to repair orchestration.

For these reasons, epithelial cells should be regarded as central intermediaries in macrophage–ILC communication rather than passive targets of immune activity. Their condition determines the tone, structure, and success of the broader innate immune network in ALI. Recognizing this triadic relationship strengthens the argument that acute lung injury is fundamentally a disease of tissue-level circuit disruption, in which immune dysfunction and structural damage are inseparable components of the same pathobiological process.

### 6.4 From coordinated repair to dysregulated circuit collapse

In a successfully recovering lung, macrophages, ILCs, and epithelial cells do not act as isolated responders but as a coordinated system that couples injury sensing to inflammatory control and tissue restoration. Early danger detection is followed by appropriate leukocyte recruitment, containment of damage, clearance of dying cells, induction of epithelial support programs, and gradual re-establishment of niche homeostasis (169–171). In this setting, macrophage–ILC–epithelial interactions form a functional repair circuit. The value of this concept lies in showing that resolution is not merely the absence of continued injury, but the presence of organized intercellular cooperation. This coordinated state, however, is inherently fragile. Acute lung injury places extraordinary stress on the alveolar niche, and the signals that initially promote defense can, when excessive or prolonged, destabilize the very circuitry required for recovery. Recruited inflammatory macrophages may dominate over resident reparative populations, epithelial cells may lose their capacity to provide coherent niche signals, and ILC programs that ordinarily support barrier restoration may become insufficient or misdirected. When these changes occur together, the system no longer behaves as a repair circuit. Instead, it enters a state of dysregulated circuit collapse in which inflammatory persistence, defective clearance, and structural vulnerability reinforce one another. This collapse is not a single event but a progressive systems-level failure. The earliest stages may involve mistimed macrophage state transitions or incomplete induction of ILC-associated tissue support. As injury persists, the burden of apoptotic and damaged cells increases, epithelial instruction deteriorates, and reciprocal immune support becomes less effective. Eventually, the network may lose its ability to switch from inflammatory amplification to reparative coordination. At that point, even if the original trigger is attenuated, the lung remains trapped in a pathologic state maintained by its own internal dysfunction. This helps explain why severe ALI can persist despite control of the initiating insult and why recovery often depends on more than suppression of early inflammatory mediators.

The concept of circuit collapse also offers a useful bridge between acute pathology and long-term outcomes. A lung that fails to restore coordinated macrophage–ILC–epithelial communication is not simply delayed in recovery; it may become predisposed to chronic epithelial fragility, prolonged immune dysregulation, fibroproliferative remodeling, or other sequelae of incomplete repair. Thus, failed recovery should be interpreted as the biological consequence of unresolved circuit dysfunction rather than the passive residue of prior inflammation. This interpretation adds mechanistic depth to the clinical observation that some patients experience persistent abnormalities long after acute respiratory failure has improved. Importantly, this model also clarifies why a purely anti-inflammatory therapeutic strategy may be insufficient. Once the repair circuit has collapsed, the major problem is no longer just excessive inflammation, but the inability of the tissue to rebuild the communication networks necessary for restoration. Effective intervention may therefore require simultaneous reprogramming of macrophages, support of ILC-associated repair pathways, and recovery of epithelial niche function. In other words, the therapeutic goal shifts from blocking inflammatory output to rebuilding circuit competence.

**Figure 4:**
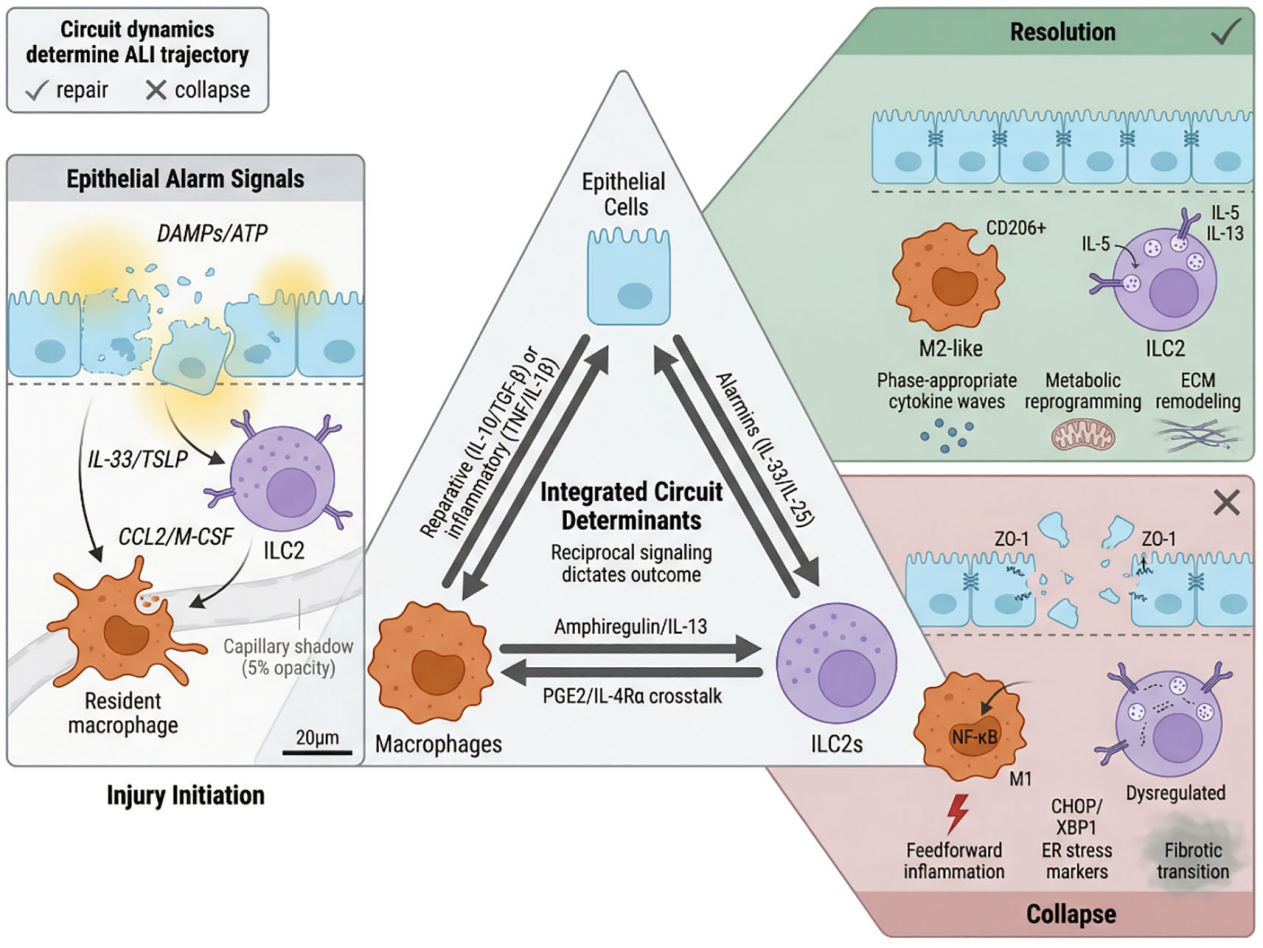
Macrophage–ILC crosstalk determines repair versus circuit collapse in acute lung injury. In ALI, epithelial injury initiates a triadic communication network among macrophages, ILC2s, and epithelial cells. When properly coordinated, this innate immune circuit supports inflammatory control, epithelial repair, and restoration of homeostasis. When dysregulated, reciprocal signaling collapses into persistent inflammatory amplification, defective tissue support, and failed repair.

Taken together, the transition from coordinated repair to dysregulated circuit collapse captures the central pathobiological logic of this review. Acute lung injury is not simply severe inflammation occurring in the lung; it is the failure of a tissue-specialized innate immune system to maintain the sequence of responses required for return to homeostasis. Macrophages, ILCs, and epithelial cells stand at the core of this system. Whether they remain functionally coordinated or fall into reciprocal dysfunction determines whether the injured lung recovers or progresses toward persistent damage and failed repair. **Table 3** presents a conceptual model in which macrophage–ILC–epithelial interactions determine whether ALI progresses toward coordinated repair or dysregulated circuit collapse. **Figure 4** summarizes the concept that macrophages, ILC2s, and epithelial cells form an integrated innate immune circuit that governs the trajectory of ALI.

**Table 3:** A conceptual framework of macrophage–ILC–epithelial circuits in acute lung injury: from inflammatory amplification to failed repair.

Circuit component	Early injury phase	Escalating injury phase	Resolution/repair phase	Failed repair phase	Therapeutic implication
Epithelial cells	Sense damage and release alarm signals	Sustain inflammatory signaling, barrier disruption, extracellular vesicle release	Re-establish niche signals and support regenerative programs	Persistent dysfunction impairs immune coordination and barrier restoration	Restore epithelial niche integrity and repair-supportive signaling
Resident alveolar macrophages	Act as frontline sentinels and initiate local responses	May amplify barrier damage if excessively activated	Transition toward efferocytosis, inflammatory control, and epithelial support	Loss or dysfunction weakens resolution and niche recovery	Preserve or reprogram resident macrophages toward reparative states
Monocyte-derived macrophages	Begin recruitment from circulation	Drive inflammatory escalation, cytokine production, and tissue injury	Can be restrained or redirected by reparative cues	Persistent dominance sustains non-resolving inflammation	Limit pathogenic recruitment or redirect toward pro-resolving phenotypes
ILC2s/ILC-associated programs	Sense epithelial stress and respond rapidly to tissue signals	May become insufficient or distorted in a highly inflammatory niche	Promote epithelial support, homeostasis, and tissue-adaptive repair	Inadequate repair signaling contributes to persistent structural vulnerability	Reinforce tissue-protective ILC-associated repair programs
Macrophage–ILC crosstalk	Limited but emerging local coordination	Context-dependent shaping of inflammatory bias	Reciprocal support promotes resolution and repair competence	Circuit collapse leads to mistimed or ineffective recovery responses	Rebuild beneficial macrophage–ILC communication networks
Overall tissue outcome	Controlled danger sensing	Inflammatory amplification and barrier breakdown	Resolution, epithelial regeneration, and homeostatic recovery	Persistent inflammation, defective clearance, and failed repair	Shift treatment from broad suppression to circuit rebalancing

## 7 Single-cell and systems-level insights into innate immune circuits in ALI

### 7.1 High-dimensional profiling redefines macrophage heterogeneity

Recent advances in high-dimensional immune profiling have substantially reshaped our understanding of macrophage biology in acute lung injury. Earlier models often treated pulmonary macrophages as a relatively uniform compartment whose function varied primarily according to the intensity of pro- or anti-inflammatory activation. This view has become increasingly inadequate. Techniques such as CyTOF and single-cell RNA sequencing have revealed that macrophage populations in ALI and ARDS are far more heterogeneous than previously appreciated, encompassing distinct subsets with different transcriptional programs, functional biases, and potential contributions to either injury propagation or repair (172). This shift is not merely technical; it has important conceptual consequences. Once macrophage diversity becomes visible at higher resolution, it becomes clear that the relevant biological question is no longer whether macrophages are activated, but which macrophage states emerge, when they emerge, and how they relate to disease stage and tissue context. Some subsets appear associated with early inflammatory amplification and leukocyte recruitment, whereas others are more closely linked to apoptotic cell clearance, epithelial support, or pro-resolving functions. Such findings reinforce the broader argument of this review that macrophage biology in ALI is fundamentally dynamic, lineage-sensitive, and tissue-dependent.

Another important contribution of high-dimensional profiling is that it exposes the coexistence of resident and recruited macrophage programs within the same injured lung. This helps explain why global descriptions of “macrophage activation” are often biologically uninformative. The lung may simultaneously contain resident alveolar macrophages with partially preserved homeostatic features, inflammatory monocyte-derived macrophages driving tissue damage, and transitional populations in the process of rewiring toward repair. Disease trajectory may therefore depend not on a dominant macrophage phenotype in the abstract, but on the evolving proportions, localization, and functional coupling of these distinct states. High-dimensional profiling makes these relationships more tractable and helps move the field beyond oversimplified polarization schemes. These approaches also align well with the emerging concept of tissue-driven inflammation. Single-cell analyses do not simply catalog immune subsets; they provide insight into how tissue damage restructures the immune ecosystem. In ALI, changes in epithelial state, chemokine gradients, and metabolic stress likely reshape macrophage composition and functional identity over time (173, 174). High-dimensional studies therefore offer an opportunity to connect cellular heterogeneity with the tissue niche that generates it. This is especially important for a circuit-based model of disease, because communication networks can only be understood if the relevant cellular participants are first defined with sufficient resolution.

At the same time, these datasets should be interpreted with care. Cellular heterogeneity revealed by transcriptomic or phenotypic clustering does not automatically translate into distinct functional entities, and many inferred macrophage states remain to be validated experimentally. Even so, the broader message is clear: macrophage heterogeneity in ALI is real, biologically meaningful, and central to understanding both injury progression and recovery. High-dimensional profiling has therefore not simply refined an existing model; it has forced a rethinking of how pulmonary innate immunity is organized.

### 7.2 Opportunities and limitations of single-cell inference for ILC–macrophage circuits

Single-cell and systems-level approaches have also created new opportunities to study interactions between macrophages and ILCs in the injured lung. By resolving cell states at high resolution and enabling computational inference of ligand–receptor communication, these methods make it possible to move beyond descriptive cell abundance and toward a more network-oriented view of pulmonary immunity. For a topic such as macrophage–ILC crosstalk, where direct functional evidence remains relatively limited, this is especially valuable. Single-cell datasets can identify candidate communication hubs, suggest phase-specific interactions, and reveal how structural and immune compartments are linked during injury and repair. This is particularly relevant because ILC populations are comparatively rare and can be difficult to analyze with conventional bulk approaches. Single-cell methods provide a way to detect ILC-associated states, situate them within the broader immune ecosystem, and examine how their transcriptional programs change alongside those of macrophages and epithelial cells. In principle, such analyses can reveal whether protective ILC-associated pathways emerge together with reparative macrophage programs, whether inflammatory macrophage dominance correlates with loss of tissue-supportive ILC signals, and how the surrounding niche shapes these relationships. This systems-level perspective is highly compatible with the circuit-based framework proposed in this review.

However, there are also clear limitations. Computationally inferred communication does not establish functional causality, and ligand–receptor pairing alone cannot determine whether a predicted interaction is biologically dominant, phase-specific, or therapeutically actionable. In addition, single-cell datasets often underrepresent rare populations, may lack sufficient temporal depth, and usually provide only partial information about spatial context. For ILC–macrophage circuits, these limitations are especially important because the meaning of a given interaction is likely to depend heavily on location within the lung, stage of injury, and concurrent epithelial damage (24, 175, 176). Without these layers of context, inferred networks remain hypotheses rather than demonstrated mechanisms. Another challenge is that many single-cell analyses provide snapshots rather than trajectories. Yet both macrophage and ILC biology in ALI are intrinsically time-dependent. A communication module that appears prominent at one time point may be absent, reversed, or functionally reinterpreted at another. Because repair failure is likely driven by mistimed or incomplete transitions, temporally static inference may miss the most biologically significant aspect of the system. This makes longitudinal and multi-timepoint designs especially important for future studies of innate immune circuits in lung injury.

Despite these caveats, the value of single-cell inference remains substantial. These methods are particularly powerful for generating mechanistic hypotheses, prioritizing cell states for functional testing, and revealing previously unrecognized organizational features of the injured lung. In that sense, they do not replace experimental immunology but complement it. For macrophage–ILC circuits, the most productive path forward will likely involve integrating single-cell discovery with lineage tracing, *in vivo* perturbation, and spatially resolved validation. This combined strategy offers the best chance of moving from inferred crosstalk to experimentally grounded models of tissue-level immune regulation in ALI.

### 7.3 Toward a spatial and temporal map of repair failure

Ultimately, the most important unanswered questions in acute lung injury may not concern which cells are present, but where they are positioned, when they interact, and how their relationships change as the tissue moves toward recovery or collapse. This is why future progress in the field will likely depend on building a spatial and temporal map of repair failure. Such a map would go beyond static descriptions of inflammatory cell infiltration and instead define the evolving architecture of macrophage, ILC, epithelial, and stromal interactions across the course of injury. A spatially informed perspective is essential because the lung is not a uniform organ. Different microenvironments within the injured lung may support very different immune states. Cells located in relatively preserved alveolar regions may participate in effective repair, whereas cells in heavily damaged, edematous, or mechanically stressed regions may become trapped in pro-inflammatory or dysfunctional programs. Without spatial resolution, these local differences are easily obscured. Yet they may be precisely what determines whether innate immune circuits remain coordinated or collapse into self-sustaining pathology. Temporal resolution is equally important. Repair failure is not a fixed endpoint but a process that emerges through cumulative miscoordination over time. Early inflammatory amplification, delayed macrophage rewiring, insufficient ILC-associated epithelial support, and collapse of structural niche signaling may each contribute at different stages. Understanding how these events are sequenced will be critical for identifying the windows during which intervention is most likely to succeed. A therapy that is beneficial during early inflammatory escalation may be ineffective or harmful once the tissue has entered a state of failed repair, and vice versa. The goal, therefore, should be to move toward a systems immunology of ALI that is explicitly spatial, temporal, and functional. Such an approach would allow investigators to define not only the components of innate immune circuits, but also the rules by which those circuits are assembled, destabilized, and potentially restored. This would represent a major step forward from current frameworks centered mainly on inflammatory intensity or endpoint pathology. It would also provide a more rational basis for therapeutic design, especially for interventions aimed at rebuilding repair competence rather than merely suppressing inflammation. In this sense, a spatial and temporal map of repair failure is not simply a technical ambition. It is the natural next step required by the conceptual shift advanced in this review. If ALI is fundamentally a disease of dysregulated tissue immune circuits, then understanding it will require defining how those circuits are organized in real time within the injured lung.

## 8 Therapeutic opportunities targeting macrophage–ILC circuits in ALI

### 8.1 Rebalancing inflammatory versus reparative macrophage states

The recognition that macrophages can either amplify injury or coordinate recovery has important therapeutic implications for acute lung injury. Rather than viewing macrophages as a uniformly pathogenic population to be suppressed, a more useful strategy is to consider how the balance between inflammatory and reparative macrophage states might be selectively reprogrammed. This is especially important in ALI, where the same broad cell lineage may contribute to early damage yet remain indispensable for later resolution and tissue repair (177–180). A therapeutic approach based solely on indiscriminate inhibition risks disrupting the very cellular programs needed for recovery. From this perspective, the therapeutic goal is not simply to reduce macrophage activation, but to restore phase-appropriate macrophage function. During the injurious phase, this may involve limiting excessive inflammatory amplification, restraining the persistence of highly pathogenic monocyte-derived macrophage programs, or reducing macrophage-mediated disruption of epithelial and endothelial barrier integrity. During the recovery phase, by contrast, the objective shifts toward enhancing efferocytosis, supporting resident alveolar macrophage recovery, and promoting the emergence of pro-resolving and tissue-supportive macrophage states. This temporal distinction is crucial. The same intervention may be beneficial or detrimental depending on whether it is delivered during escalating inflammation or during attempted tissue restoration. This framework suggests several therapeutic principles. One is that resident alveolar macrophages and recruited monocyte-derived macrophages should not necessarily be targeted in the same way. Because resident macrophages are embedded in the tissue niche and may be particularly important for coordinated repair, preserving or restoring their homeostatic and reparative capacity may be advantageous. In contrast, excessive accumulation or persistence of inflammatory monocyte-derived macrophages may sustain injury and delay resolution. Therapies that selectively attenuate the harmful dominance of recruited inflammatory macrophages while preserving reparative macrophage competence would therefore fit well with a circuit-based model of ALI.

Another important principle is that macrophage reprogramming may be more effective than macrophage depletion. Approaches that enhance apoptotic cell clearance, improve responsiveness to pro-resolving tissue signals, or reinforce functional transitions toward epithelial support may offer advantages over broadly anti-inflammatory strategies. The recent emphasis on efferocytosis and trained pro-resolving macrophage states illustrates this point well. Such findings suggest that macrophages are not merely targets to suppress, but therapeutic substrates that can potentially be instructed toward recovery-promoting functions. At a broader level, this approach reflects a shift in critical care immunology from blunt inflammatory control toward restoration of tissue-adapted immune competence. In ALI, the key question is not only how to reduce damage, but how to help the lung regain its capacity for organized recovery. Because macrophages are central coordinators of this transition, strategies that rebalance their states may become one of the most promising routes toward more effective intervention. The challenge, however, will be to define biomarkers, time windows, and tissue contexts that allow such reprogramming to be applied with precision rather than in a one-size-fits-all manner.

### 8.2 Enhancing ILC-associated tissue repair programs

If macrophage-directed therapies aim to restore inflammatory control and reparative coordination, then ILC-associated strategies may offer a complementary route to support epithelial recovery and tissue homeostasis. This possibility is particularly relevant for ILC2s, which occupy a strategic position at the interface between epithelial alarm sensing and tissue-protective output. In acute lung injury, where barrier integrity and regenerative capacity are major determinants of outcome, enhancing endogenous ILC-associated repair programs may help shift the lung away from persistent structural vulnerability and toward functional restoration. The rationale for this approach lies in the observation that ILC2s are capable of producing mediators linked to epithelial maintenance, tissue protection, and barrier repair (181). These functions suggest that ILC2s may contribute to the endogenous regenerative machinery of the lung, especially in phases when adaptive immunity is too slow or too antigen-restricted to provide immediate support. Therapeutic enhancement of such pathways could therefore help sustain epithelial survival, improve the quality of repair, and reduce the likelihood that residual structural injury continues to perpetuate local inflammation. However, translating this concept into therapy requires careful nuance. ILC biology is strongly context dependent, and broad enhancement of ILC activity would not necessarily be beneficial in every phase or form of lung injury. Depending on the surrounding milieu, ILC-associated pathways may also contribute to inappropriate immune bias or pathological remodeling. Thus, the therapeutic objective should not be framed as simple expansion or activation of ILC2s in general. Rather, it should focus on selectively reinforcing those ILC-associated programs that are most closely linked to epithelial support, barrier adaptation, and restoration of homeostasis. This distinction mirrors the therapeutic logic already discussed for macrophages: the aim is to recover functionally beneficial states rather than indiscriminately manipulate cell numbers. In practical terms, that may mean promoting tissue-protective outputs such as amphiregulin-associated repair, strengthening the epithelial signals that support constructive ILC responses, or preserving the niche conditions under which ILCs contribute to recovery rather than maladaptation. Such strategies would align well with a circuit-based understanding of ALI, in which successful repair depends on reinforcing endogenous communication networks rather than merely blocking inflammatory endpoints.

Another important implication is that ILC-targeted approaches may be especially valuable when integrated with macrophage-directed strategies. Because ILCs help shape the quality of local tissue adaptation and may also influence macrophage functional bias, supporting ILC-associated repair programs could enhance the likelihood that macrophages transition into states favorable for resolution. In this sense, ILCs may serve as amplifiers of restorative network behavior rather than isolated therapeutic endpoints. Their value may therefore lie not only in their direct effects on epithelial cells, but also in their capacity to reinforce a broader reparative circuit within the injured lung.

### 8.3 Targeting tissue-derived signals and intercellular communication hubs

A major implication of the circuit-based model proposed in this review is that therapeutic intervention in ALI may be more effective when directed at communication hubs rather than isolated terminal mediators. Traditional anti-inflammatory strategies have often focused on blocking single cytokines or broadly suppressing immune activation. While such approaches may reduce parts of the inflammatory cascade, they do not necessarily restore the tissue-level coordination required for repair. If ALI is fundamentally a disease of disrupted intercellular signaling, then targeting the hubs that organize macrophage, ILC, and epithelial communication may offer a more coherent therapeutic logic. These hubs include epithelial-derived alarm signals, niche-maintaining growth factors, extracellular vesicle-mediated communication, metabolic cues, and trophic mediators exchanged between immune and structural cells. Such signals are attractive because they sit upstream of multiple downstream effects. They may influence how macrophages are activated, whether ILC-associated repair programs are initiated, and whether epithelial cells remain capable of receiving regenerative support. As a result, restoring or modulating these communication pathways could, in principle, realign multiple compartments of the injured lung simultaneously rather than altering one arm of the response in isolation. This approach also better reflects the biology of tissue recovery. Repair is not produced by a single dominant molecule, but by the coordinated interaction of many signals that together create a permissive niche for resolution. For example, the same epithelial cell that is damaged during ALI is also a source of instructive cues that sustain resident macrophage identity and activate ILC-mediated support pathways. Likewise, macrophage-derived mediators can influence not only inflammation but epithelial proliferation and the regulation of inflammatory monocyte-derived populations. Communication hubs therefore represent leverage points within a complex network. Intervening at these nodes may be more likely to restore system behavior than simply neutralizing one inflammatory product after the circuit has already destabilized.

Targeting intercellular communication hubs also aligns naturally with emerging technological approaches. High-dimensional immune profiling, ligand–receptor inference, extracellular vesicle analysis, and spatial transcriptomics increasingly allow investigators to identify the signaling relationships most strongly associated with either successful recovery or repair failure. This may eventually make it possible to move beyond generic anti-inflammatory treatment and toward interventions that are tailored to the dominant circuit disruption present in a given phase or patient subgroup. In that sense, communication-based therapeutics could become one of the most promising translational outcomes of the tissue-immunology framework. At the same time, this strategy comes with challenges. Because communication hubs often regulate multiple processes simultaneously, they may produce context-dependent effects that are difficult to predict without precise spatial and temporal knowledge of the injury state. Nevertheless, the broader principle remains compelling: in a disease characterized by disrupted immune–structural coordination, restoring intercellular communication may be as important as suppressing inflammation itself. This marks a conceptual shift from endpoint inhibition to network repair.

### 8.4 Challenges for clinical translation

Despite the growing conceptual appeal of targeting innate immune circuits in acute lung injury, translating these ideas into clinical practice remains difficult. One major challenge is the heterogeneity of ALI itself. Patients differ in etiology, severity, timing of presentation, degree of infection control, ventilatory exposure, and baseline immune status. These differences are likely to have major effects on macrophage states, ILC activity, epithelial niche integrity, and the overall structure of innate immune circuits. As a result, interventions that are beneficial in one biological context may fail or even prove harmful in another. A second challenge is temporal instability. The immune landscape of ALI changes rapidly over time, and the same cell type may play very different roles at different stages of disease. An inflammatory macrophage program that should be restrained early may need to be preserved or redirected later. An ILC-associated pathway that supports epithelial protection in one phase may become less beneficial or differently regulated in another. This means that successful therapy will likely require not only biological specificity, but also precise timing. Static treatment models are poorly suited to a disease defined by evolving circuit dynamics. A third challenge is the difficulty of measuring tissue states in real time. Much of the mechanistic framework for macrophage–ILC–epithelial circuits comes from experimental systems, whereas in clinical settings access to the alveolar niche is limited. Peripheral blood measurements may capture some aspects of inflammatory activation but are often insufficient to define the state of the pulmonary immune network itself. This creates a translational gap: therapies may need to be directed at tissue-level dysfunction, yet the tools to identify that dysfunction at the bedside remain underdeveloped. Bridging this gap will require biomarkers that reflect not only systemic inflammation, but also local repair competence, macrophage balance, and epithelial–immune communication.

Another challenge is that circuit-based interventions are unlikely to work through simple on/off effects. Because they aim to rebalance networks rather than eliminate individual targets, their success may depend on partial modulation, combination strategies, or sequential treatment across different disease phases. This complexity is scientifically appropriate but clinically demanding. It suggests that future therapies for ALI may need to resemble ecological interventions within a damaged tissue system rather than conventional single-target pharmacology. Even so, these challenges should not be interpreted as reasons to abandon the circuit framework. On the contrary, they highlight why earlier therapeutic paradigms based largely on nonspecific inflammatory suppression have had limited success. The failure to fully account for tissue context, timing, and intercellular coordination may itself be part of the reason effective therapies remain elusive. A more nuanced understanding of innate immune circuits therefore does not complicate the problem unnecessarily; it may instead be necessary to solve it.

## 9 Conclusion and future perspectives

Acute lung injury is no longer adequately explained as a syndrome of uncontrolled inflammation alone. Although cytokine excess, neutrophil infiltration, and alveolar–capillary barrier disruption remain central features of disease, they do not fully account for the marked heterogeneity in recovery, the persistence of pulmonary dysfunction in severe cases, or the transition from acute injury to failed repair. A more integrated view is needed, one that recognizes the lung as a tissue-specialized immune environment in which structural and innate immune cells continuously shape one another’s behavior. Within this framework, macrophages emerge as key determinants of disease trajectory because their functional plasticity governs whether the injured lung remains trapped in inflammatory escalation or begins to move toward resolution. Resident alveolar macrophages, recruited monocyte-derived macrophages, and transitional macrophage states together create a dynamic myeloid landscape whose biological significance depends on timing, niche context, and capacity for rewiring. At the same time, ILCs, particularly ILC2s, provide an additional layer of tissue-adapted innate regulation by linking epithelial alarm sensing to barrier support, homeostatic restoration, and modulation of the broader immune environment. Their roles in ALI underscore that recovery is not simply the dampening of inflammation, but the active reconstruction of coordinated tissue immunity. A central conclusion of this review is that macrophages, ILCs, and epithelial cells should be understood not as independent actors, but as components of an integrated innate immune circuit. In the successfully recovering lung, this circuit couples danger sensing to apoptotic cell clearance, inflammatory control, epithelial support, and niche restoration. In severe or non-resolving injury, however, these interactions become dysregulated. The result is not merely persistent inflammation, but collapse of the intercellular coordination required for repair. In this sense, failed recovery after ALI is best interpreted as a state of circuit dysfunction rather than a simple extension of acute damage. This perspective has important implications for future research. First, greater emphasis should be placed on defining the spatial and temporal organization of innate immune circuits in the injured lung. It will not be enough to identify which cells are present; it will be necessary to determine where they interact, when they change state, and how those interactions influence progression toward resolution or collapse. Second, the field must move from descriptive heterogeneity to mechanistic validation. Single-cell and systems-level approaches have revealed a rich landscape of macrophage and ILC states, but the causal roles of many predicted interactions remain to be established *in vivo*. Third, translational efforts should increasingly focus on restoring repair competence rather than simply suppressing inflammatory output. This means identifying biomarkers of tissue-level circuit failure and developing interventions that can re-establish macrophage rewiring, ILC-associated repair programs, and epithelial–immune reciprocity. Ultimately, the study of ALI is entering a phase in which tissue immunology, systems biology, and translational critical care must be brought into closer alignment. Macrophage plasticity, ILC crosstalk, and epithelial niche signaling provide a powerful conceptual framework for doing so. By understanding acute lung injury as a disorder of dysregulated innate immune circuits, it becomes possible to explain both inflammatory amplification and failed repair within a single model. This integrated view may not only improve mechanistic understanding, but also open the way to a new generation of therapies aimed at rebuilding the capacity of the injured lung to return to homeostasis.
